# Rhythmic Manipulation of Objects with Complex Dynamics: Predictability over Chaos

**DOI:** 10.1371/journal.pcbi.1003900

**Published:** 2014-10-23

**Authors:** Bahman Nasseroleslami, Christopher J. Hasson, Dagmar Sternad

**Affiliations:** 1 Department of Biology, Northeastern University, Boston, Massachusetts, United States of America; 2 Department of Physical Therapy, Movement and Rehabilitation Sciences, Northeastern University, Boston, Massachusetts, United States of America; 3 Department of Electrical and Computer Engineering, Northeastern University, Boston, Massachusetts, United States of America; 4 Department of Physics, Northeastern University, Boston, Massachusetts, United States of America; 5 Center for the Interdisciplinary Research on Complex Systems, Northeastern University, Boston, Massachusetts, United States of America; University College London, United Kingdom

## Abstract

The study of object manipulation has been largely confined to discrete tasks, where accuracy, mechanical effort, or smoothness were examined to explain subjects' preferred movements. This study investigated a rhythmic manipulation task, which involved continuous interaction with a nonlinear object that led to unpredictable object behavior. Using a simplified virtual version of the task of carrying a cup of coffee, we studied how this unpredictable object behavior affected the selected strategies. The experiment was conducted in a virtual set-up, where subjects moved a cup with a ball inside, modeled by cart-and-pendulum dynamics. Inverse dynamics calculations of the system showed that performing the task with different amplitudes and relative phases required different force profiles and rendered the object's dynamics with different degrees of predictability (quantified by Mutual Information between the applied force and the cup kinematics and its sensitivity). Subjects (n = 8) oscillated the virtual cup between two targets via a robotic manipulandum, paced by a metronome at 1 Hz for 50 trials, each lasting 45 s. They were free to choose their movement amplitude and relative phase between the ball and cup. Experimental results showed that subjects increased their movement amplitudes, which rendered the interactions with the object more predictable and with lower sensitivity to the execution variables. These solutions were associated with higher average exerted force and lower object smoothness, contradicting common expectations from studies on discrete object manipulation and unrestrained movements. Instead, the findings showed that humans selected strategies with higher predictability of interaction dynamics. This finding expressed that humans seek movement strategies where force and kinematics synchronize to repeatable patterns that may require less sensorimotor information processing.

## Author Summary

Daily actions frequently involve manipulation of tools and objects, such as stirring soup with a spoon or carrying a cup of coffee. Carrying the cup of coffee without spilling can be challenging as the coffee can only be indirectly controlled via moving the cup. Interaction forces between the cup and the coffee can be complex and, due to the nonlinear dynamics, lead to chaotic behavior. If unpredictable, an individual has to continuously react and compensate for the unexpected behavior. We hypothesized that when interacting continuously with such a complex object, humans seek a strategy that keeps the object's behavior predictable to avoid excessive sensorimotor processing. Using a simplified model of the cup of coffee, we analyzed forces and their relation to hand movements for rhythmic manipulation. Subjects practiced the modeled task in a virtual set-up for 50 trials. Experimental results show that subjects establish repeatable and predictable patterns. Importantly, the experiment was set up such that this increase in predictability was paralleled by an increase in exerted forces and a decrease in smoothness. Hence, this result showed that in complex object manipulation humans did not prioritize mechanical efficiency, but sought predictable interactions.

## Introduction

The behavioral repertoire of humans includes a wide variety of movements in interaction with objects and the environment. Grasping and lifting a book involves manual interaction with a rigid object and turning a key in a keyhole involves moving a rigid object against a kinematic constraint. Such actions and interactions become particularly intriguing when the objects themselves have internal degrees of freedom and thereby add complex dynamics to the interaction. Bringing a cup of coffee to one's mouth is one such example: while one moves the cup, the coffee is only indirectly controlled via moving its container. The dynamics of the sloshing coffee creates complex interaction forces that the person has to take into account during control [1], [2]. Even though such complex manipulation of objects and tools are ubiquitous in human behavior, our understanding of how humans achieve dexterity in complex object manipulation tasks is still limited.

Given the large number of motor neuroscience studies on goal-directed movements, relatively few have investigated the manipulation of complex objects. A frequently examined task utilizes the classic control problem of balancing a pole, where the task of the controller is to stabilize an inherently unstable system. Several studies analyzed human trajectories during balancing to infer properties of the controller, such as intermittent, continuous, or predictive control with forward or inverse models [3], [4]. For instance, mathematical modeling of the controller [5] and nonlinear time-series analysis of the trajectory [6] have been used to study the role of noise and delays to eventually distinguish between the continuous or intermittent nature of control in pole balancing tasks [7], [8]. Another line of study examined the task of compressing a buckling spring and focused on multisensory integration with different time delays, and modeled task performance with a simple model displaying a subcritical pitchfork bifurcation [9]. A final line of studies examined a positioning task, where the subject placed a mass attached to a spring to a target. These latter studies posited optimization criteria, such as generalized kinematic smoothness [10], minimum effort and maximum accuracy [11], minimum acceleration with constraints on the center of mass [12], or task-specific criteria such as the maintenance of adequate safety margins in carrying a virtual cup of coffee [1].

Given the variety of these theoretical approaches, it has proven difficult to arrive at a converging answer about the nature of the human controller in such complex tasks. Probably, not only the many unknowns in the human control system and the magnitude and type of noise and delays, but also the complex challenges of the task itself may have posed hurdles to converge at an answer. Given these problems, the present study pursued an approach that makes no assumptions about the human controller. Rather, we examine the dynamics of the task and the challenges and opportunities it poses to the human controller. Using a simplified model of a “cup of coffee”, and implementing it in a virtual environment, we characterize the range of possible object behaviors for a desired task performance. In simulations, we can analyze the space of all performances or strategies that satisfy the task and evaluate them with respect to different criteria. This approach is consistent with previous work of our group on simpler tasks, such as throwing or bouncing a ball, where performance variability was analyzed against the fully known solution space [13], [14].

It is noteworthy that all previous studies on complex object manipulation examined movements that are essentially discrete or consist of a sequence of discrete movements. Therefore, the criteria that describe the preferred manipulation strategies, e.g. smooth object transport [10], accuracy and effort [11], and safety margins [1], may not generalize to rhythmic movements. The present study examined continuous rhythmic interactions with a complex object, similar to sewing, rowing, or rhythmic machinery operation. It is important to point out, that in complex nonlinear object interaction, continuous rhythmic movements face very different challenges from discrete movements. The continuity of the interaction allows the nonlinear characteristics to emerge and give rise to complex chaotic or unpredictable behavior. As extensively discussed in the literature of nonlinear dynamics [15], [16], small changes in (initial) states and in parameters of a nonlinear system can dramatically change the long-term behavior of the system [17], leading to quasi-periodic or chaotic patterns. Given the potential emergence of chaotic or unpredictable behavior in the manipulation of nonlinear objects, how does this affect humans' motor strategies? Answering this question can be highly challenging, as the interaction with unpredictable dynamics may yield extremely variable behavior, which makes the analysis and interpretation onerous. This study attempted to provide an approach and methods to deal with this challenge.

We expect that the complexity and predictability of the object's dynamics play a dominant role in shaping the performer's movement strategy. Note that while chaotic behavior is predictable in the strict mathematical sense, in most cases it is unpredictable for a human in practical terms. We hypothesized that humans avoid chaotic or “unpredictable” solutions and favor movements that keep the object's behavior predictable, thereby simplifying the interactions with the object. If the potential for unpredictability is high, such as in continuous manipulation of a complex object, we hypothesize that subjects will prioritize predictability over other criteria, such as minimizing mechanical force or effort [18]–[20], kinematic smoothness [10], [21], or other criteria. Note that predictability of object dynamics affects the amount of required information processing, unlike other efficiency criteria, such as force, that tax energy processing in the human system.

The present study was designed to investigate the role of the object's predictability in rhythmic manipulation of an object with complex dynamics. We chose a model task that mimics the action of carrying a cup of coffee: moving a cart with a suspended pendulum. Previous work of our group developed a virtual implementation of a simple model in two dimensions and examined movement strategies in discrete displacements [1], [22]. This study used the same model, but now asked subjects to oscillate the “cup of coffee” between two targets. To derive quantitative predictions for humans' preferred strategies, we use inverse dynamics on the model to simulate the space of all movement strategies that achieved the task. We proceed by analyzing human movements and compare them to the set of all solutions. Thereby, we can identify possible strategies in a rhythmic object manipulation task. If subjects prioritize predictability over other criteria, we expect it to better identify and explain the human control strategies.

## Methods

### Model Analysis and Predictions

#### 2.1.1. Mathematical model of the object and the task

**Figure 1:**
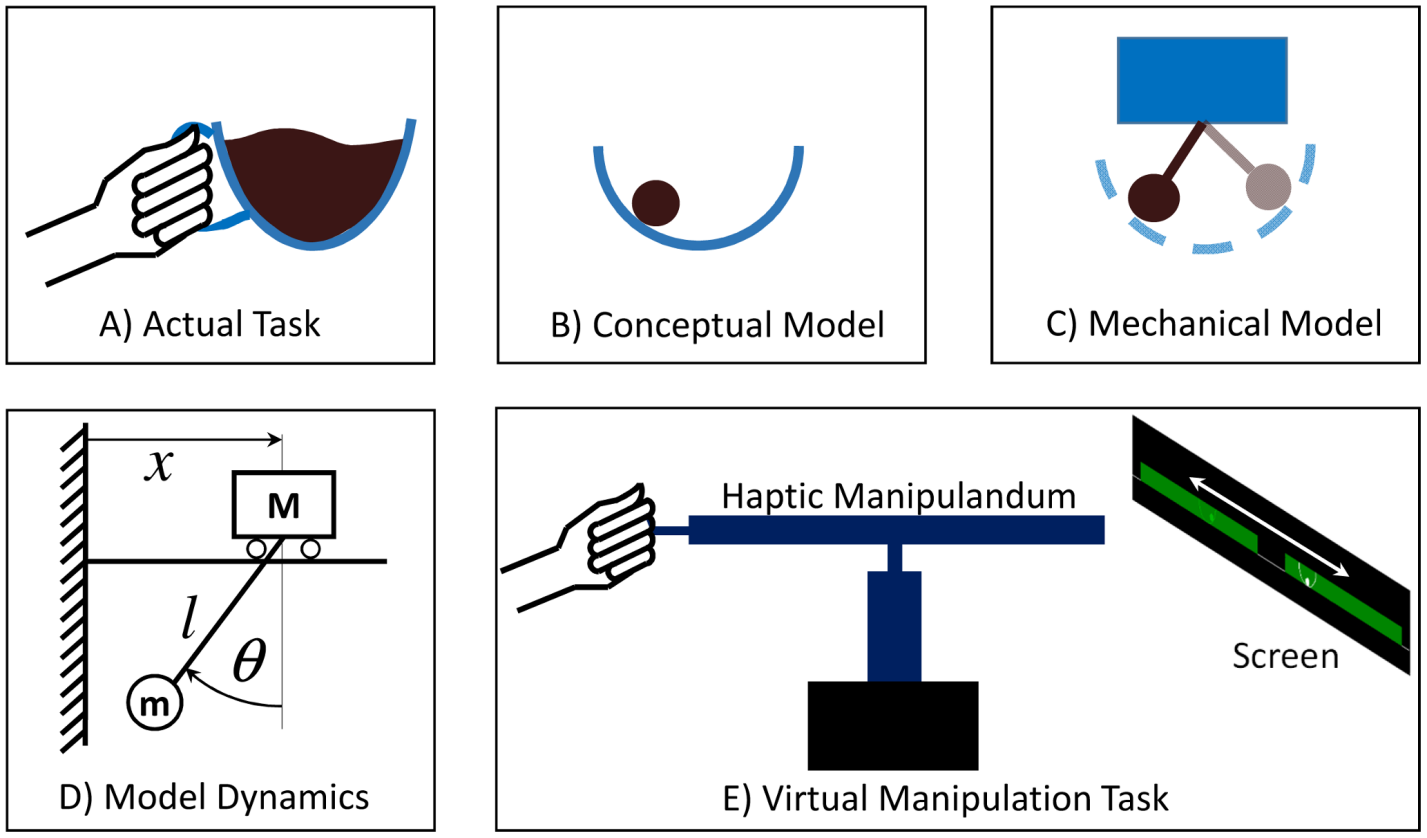
Model of the task and experimental setup. **A**: The actual task of manipulating a cup of coffee. **B**: Conceptual model of a cup of coffee as a ball in a cup. **C**: Mechanical model of the cart-and-pendulum used as a simplified two-dimensional model for the ball-in-the-cup system. **D**: Model dynamics. **E**: Task performed in the virtual environment: the haptic manipulandum provides the real-time mechanical interaction with the object; the behavior of the system is displayed on a projection screen.

The task of moving a cup of coffee was simplified to that of moving a cup with a ball inside, representing the complex dynamics of the coffee (Figure 1). The cup was reduced to an arc in 2D and the cup's motion was confined to one horizontal dimension 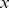. The ball's motion was modeled by a pendulum suspended from the cart. The arc of the cup corresponded to the ball's semi-circular path. In this simplification, the governing equations of the nonlinear dynamic system were identical to the well-known model problem of the cart-and-pendulum [23]. The cup motions were represented by the cart, modeled as a point mass that moved along the horizontal 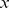
- axis. The ball was modeled as a second point mass attached to a rigid mass-less rod with one angular degree of freedom. The hand moving the cup is represented by the force 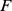 acting on the cart in the horizontal direction. This simplification maintains key features of the dynamics, while allowing an implementation that was easy to interpret [1], [2], [20]. The governing equations of the system dynamics are:
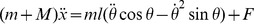
(1)

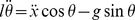
where 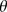, 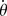 and 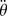 are angular position, velocity, and acceleration of the ball; 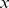, 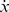 and 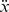 are the cart/cup position, velocity and acceleration; 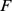 is the external force applied to the cup. Parameters of the system are 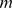 and 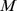 representing the masses of ball and cup, respectively; 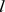 is the length of the rod (pendulum length); and 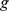 is the gravitational acceleration. For the simulations, the parameters 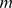, 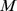, 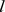 and 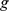 were 0.6 kg, 2.4 kg, 0.25 m and 9.81 m/s^2^, respectively. This model is equivalent to the one derived and used in previous studies [1], [20].

#### 2.1.2. Kinematic description of the task and behavior

The cup-and-ball dynamics has two mechanical degrees of freedom, which requires four state variables to fully describe the system at each time instance: 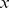, 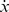, 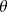, 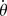. The applied force on the cup 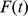 represents the input of the forward dynamics model with cup position 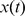 as the output. For the inverse dynamics model of the object 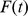 is the output. For our analysis and simulations, we did not want to assume a specific input function or a controller, *rather we wanted to infer the required input force for a kinematically defined task and strategy*. Therefore, inverse dynamics was used to solve Equation (1) to obtain the force profile 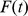 that was required to generate the oscillatory cup trajectory required by the task. For the analysis of rhythmic manipulation and inverse dynamics simulations, the full behavior was simplified to facilitate predictions.

**Figure 2:**
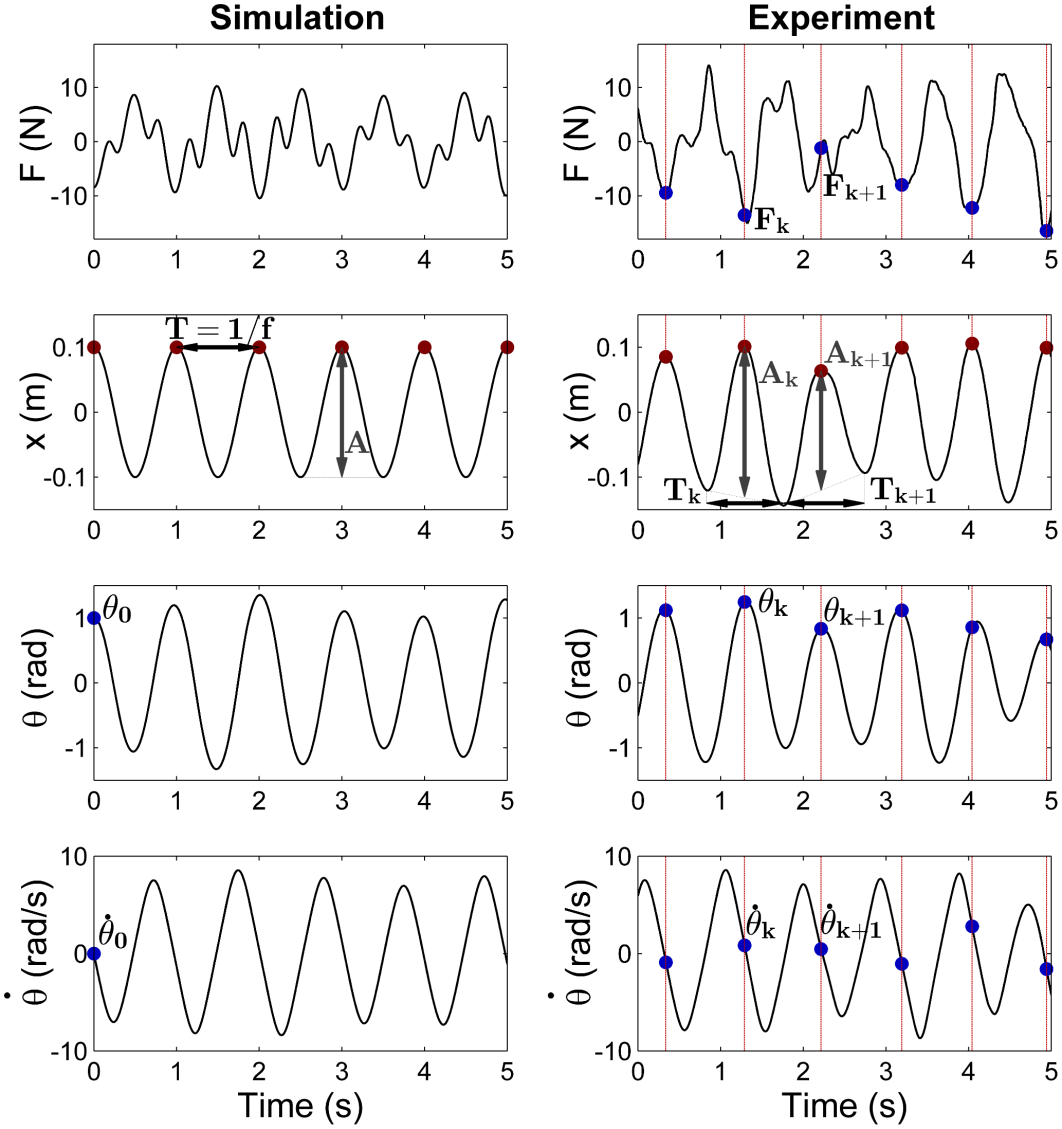
Examples of force profiles and task kinematics in simulation and experiment, with corresponding definitions of the main variables. The left column of panels shows the simulations: the cup kinematics 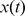 is defined as a pure sine wave with fixed amplitude 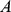 and frequency 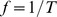; ball angle 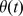 and angular velocity 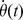 are fully defined by their initial values 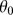 and 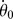. The force *F* is found by solving the inverse dynamics for these kinematic profiles (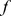 = 1.0 Hz, 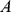 = 0.2 m, 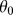 = 1.0rad, 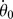 = 0 rad). The right column shows the experimental kinematics: cup position 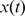, ball angle 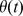 and ball angular velocity 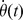 are used to estimate the frequency 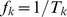, amplitude 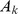, initial ball angle 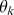, and initial ball angular velocity 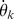 at each cycle of the trajectories. Red dots and vertical red lines indicate the location and time of the peak cup positions, at which the initial ball angle and angular velocity values for each cycle were determined, 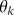 and 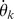, marked by blue dots. The experimental force profile is the net force applied by the subject to the manipulandum.

Given the oscillatory nature of the cup movements in the task, the trajectory of the cup 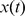 was approximated by a sine function with peak-to-peak amplitude 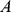 and frequency 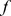: 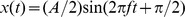, where 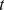 is time. Figure 2 shows simulated time series based on the sinusoidal cup trajectories. While 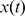 (and 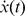) is sinusoidal by assumption, the displacement of the ball 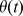 and 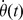 was not. The ball kinematics and the shape of the force profile, required to generate sinusoidal cup trajectories, depended on the initial values of 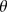 and 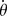 at 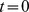. The adjacent experimental data show that the sinusoidal assumption of the cup displacement is justified. Also, the simulated and measured force profiles have similar magnitude, although with different patterns. This is partly due to the fact that the simulation was initialized at 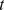 = 0 with no further modification or correction, while in the experimental data online corrections were in all likelihood present.

To facilitate predictions, we simplified the description of the model's behavior to four parameters. Assuming sinusoidal behavior, the kinematic profile of the cup 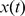 was fully described by the parameters amplitude 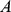 and frequency 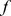. As long as no external torque or force was applied to the ball/pendulum, the cup trajectory was fully determined by the parameters amplitude 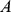 and frequency 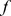; the initial values of the ball states 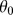 and 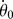 then fully specify the dynamics of the ball. This was demonstrated by numerically solving the system's differential equations, Equation (1), that gave the unique ball trajectories 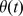 and 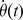. Hence, the full kinematics of the system could be represented by four scalar values 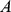, 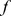, 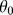 and 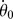, which were referred to as *execution variables*. These scalar *execution variables* spanned a 4-dimensional space of all possible task kinematics and defined the *result space*. Each point in the *result space* represents one type of system kinematics, trajectories of the ball and cup resulting from a force profile applied to the cup. Each point in result space is referred to as a *strategy*.

#### 2.1.3. Kinematic and force profiles for different task strategies

**Figure 3:**
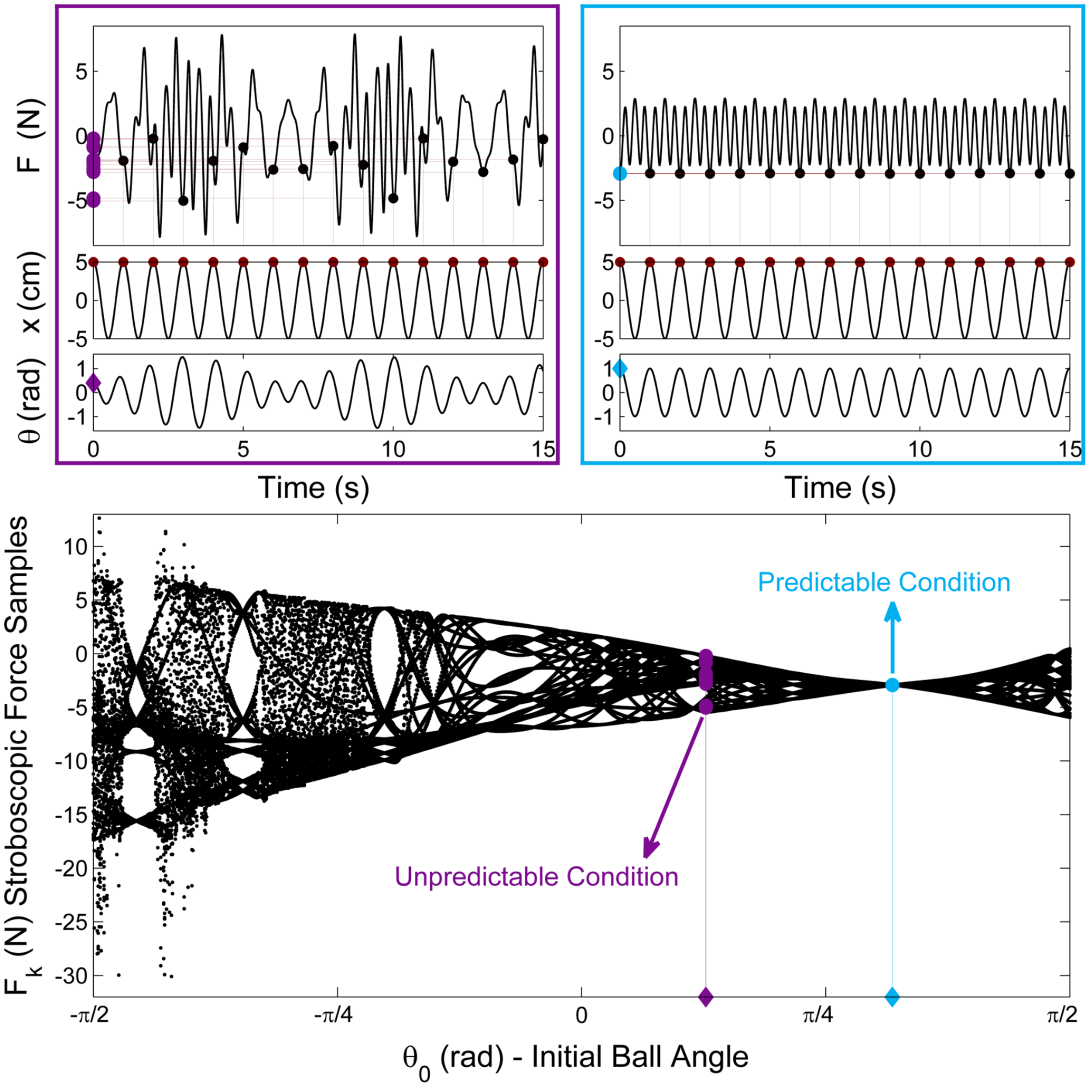
Two profiles of force and cup and ball displacement exemplify unpredictable dynamics (left) and predictable dynamics (right). The strobing procedure is illustrated by the dots and the vertical lines: at every peak of the cup displacement, the value of force is picked; the strobed force values are then projected onto the vertical axis to show the distribution for each simulation. The left example shows a scattered distribution, while the right periodic profile only shows one value. The bifurcation diagram summarizes all force distributions as a function of the parameter 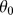, the initial ball angle. The vertical axis displays the stroboscopic samples of force values 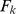, simulated from a 1.0 Hz sinusoidal cup displacement 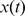 with 10 cm (full peak-to-peak) amplitude and 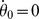
rad/s. The horizontal axis shows the initial ball angle 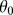. The plot shows that when the simulation starts from the initial angle 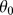 = 1.0rad, the strobed force values do not change in successive cycles (blue), corresponding to the time profile on the right. At 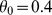
rad, there is variance in the strobed values of force 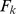, corresponding to the plot on the left (purple). The variance of 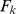 was used to define a measure for the predictability of object's dynamics (see *Predictability Index* in the supporting Text S1).

Figure 3 (top) shows two example profiles generated by inverse dynamics calculations with two different initial ball states that both result in a sinusoidal cup trajectory 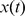. The left force profile 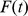 is an example for unpredictable fluctuations, while the right force profile shows a simple periodic waveform that is predictable. Both produce a sinusoidal cup displacement, but are associated with different ball displacements. To characterize and summarize the pattern of force (input) profiles with respect to the cup kinematics, i.e. the output behavior, we performed stroboscopic sampling of the force, timed by the periodic input: for each 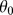, the force profiles 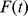 were strobed at every peak amplitude of the cup position 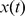 (see supporting Text S1 for more detail). The representative force and kinematic profiles in the top left figure also show the strobed force values. All strobed force values were then projected onto the 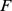-axis to yield a distribution. For this example with 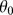 = 0.4 rad, the force values were in the range of −1.37N and −6.59N with little regularity or predictability in the pattern. In contrast, the representative time series on the top right shows a periodic solution, using 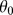 = 1.0 rad. Here, the system displays simple periodic behavior with all force values equal to −4.1N. Finally, for 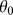 = −1.35rad, the sparse scatter of data between +8.75N and −30.0N represents highly chaotic behavior. To visualize this changing input-output relationship across different parameters, a bifurcation diagram summarized the distributions of the strobed force values as a function of the selected control parameter 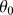 [15], [16]. The diagram shows a pattern similar to the widely discussed period-doubling behavior of complex systems. Such emergence of a complex range of behaviors from the relatively simple object/task dynamics has been widely discussed in the literature on nonlinear dynamics [5], [17], [24], [25].

While quasi-periodic and chaotic behaviors are predictable in the exact mathematical sense, they are computationally and practically unpredictable. Quasi-periodic behavior, or even periodic behavior with more than one visited value per point of input cycle (e.g. in period doubling), may appear unpredictable to the human performer. Thus, most deviations from the single fixed-value input-output relationships can be considered unpredictable from the performing subject's viewpoint.

#### 2.1.4. Predictability of the object's dynamics: Mutual Information

To characterize the predictability of the object's dynamics for each kinematically defined strategy, i.e. each point in *result space*, the relation between the simulated continuous force profile 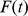 and the kinematic trajectories 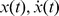 of the cup was quantified by *Mutual Information* [26]. Mutual Information is based on the statistical distributions of the time series and serves as a nonlinear measure of correlation. In the present context, it serves as a measure of the predictability of the object's dynamics as it quantifies the long-term evolution of the object's behavior based on the perfect realization of a sinusoidal cup trajectory.

To facilitate the calculation of Mutual Information of 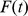 and the cup kinematics 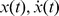, the sinusoidal cup kinematics was represented by their phase 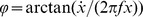. *Mutual Information* was calculated from the two-dimensional 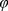-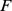 probability distributions [26]:
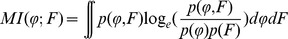
(2)where 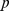 denotes the probability density functions for 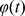 and 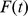. The probability density functions were estimated by linear interpolation of nonlinear Gaussian smoothing kernels, using Silverman's method for finding the parameters [27]. *MI* was a scalar measure evaluating each strategy, defined by each point of the *result space*. Using the natural logarithm, *MI* was expressed in units of *nat*.

In order to visualize the values of *MI* in the 2D result space, two of the four variables could be fixed to reduce the display to two dimensions: the frequency 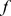 was fixed at 1.0 Hz, consistent with the experimental task (subjects were paced by a metronome at 1.0 Hz). The initial value of the angular velocity 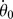 was set to zero for the 2D display. The range of 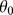 values was limited to the two horizontal positions of the pendulum corresponding to the boundaries of the displayed cup; in the experiment, subjects rarely exceeded these values. The amplitude was limited to the values that were possible in the experimental task.

**Figure 4:**
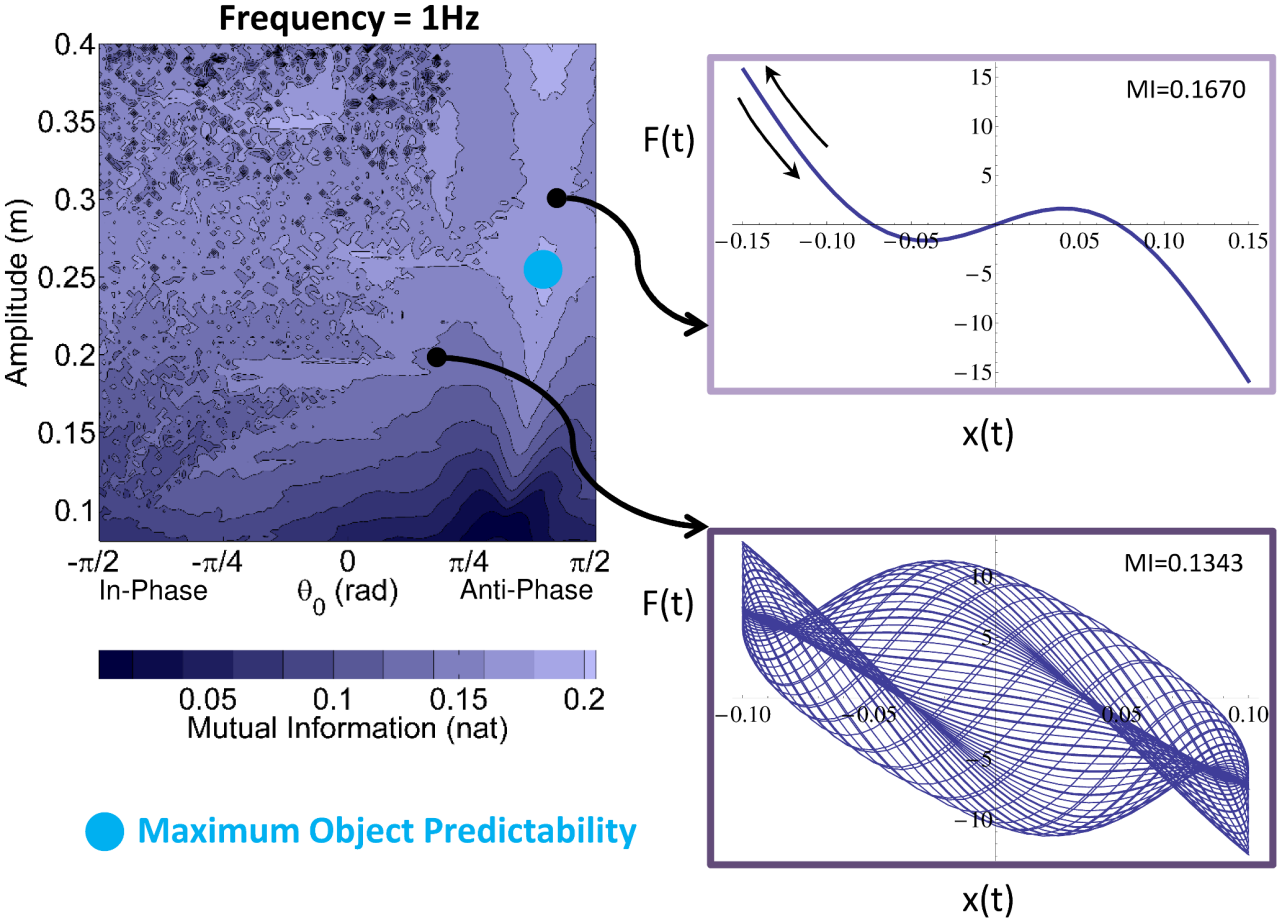
Map of *Mutual Information* in the 2D *result space* spanned by amplitude 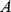 and initial ball angle 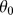 (left) and examples of force-kinematics relationship (right). The color map shows that realization of a 1.0 Hz sinusoidal cup trajectory with different amplitudes and initial angles differ in the resulting predictability of the object dynamics. *Mutual Information*, coded by color shades, describes the predictability of the object's behavior for a 1.0 Hz sinusoidal cup trajectory with different 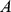 and 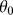. The light blue circles indicate the strategies with the highest predictability of object's dynamics. The force-cup displacement plots on the right correspond to 2 representative points (strategies) in the *result space*. The profile on the top right shows high *Mutual Information* with consistent force-kinematic relationship over successive cycles (arrows indicate the traversal on the same constant curve over consecutive cycles). The profile on the bottom right shows a strategy with low *Mutual Information*, where the force-kinematic relationship changes in every cycle.

Figures 4 (left) shows *MI* for all strategies plotted on the two-dimensional 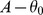 *result space*. *MI* is color-coded, with lighter shades denoting higher MI, interpreted as higher predictability. Figure 4 (top right) shows an example of a strategy with predictable object behavior and high *MI*. The force profile was plotted against the displacement of the cup and showed a consistent force-kinematic relation across successive cycles (arrows indicate the same constant curve over consecutive cycles). In contrast, the panel below shows a strategy with low *MI*: the force-kinematic relationship changes across successive cycles. The 2D result space shows connected areas with similar *MI* values, signaling that small changes do not change the value of *MI*. The top left region of the map, at negative 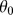 and high 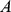 values, shows a ruggedness with small islands of *MI* values that signal chaotic regimes. On the right side, there is an extended region with high *MI* values. The blue circle denotes the location with the highest *Mutual Information*, i.e. highest predictability.

To cross-validate the quantification for predictability by *Mutual Information*, we defined a second index that quantified the scatter pattern of the strobed force values for each point in *result space*. As shown in the supporting Text S1 and Figure S1, this second *Predictability Index* rendered predictions that closely matched those of *MI*. For simplicity, we only present results from *Mutual Information*.

#### 2.1.5. Sensitivity of the object's predictability

Inspection of Figure 4 shows one more important feature: the top right side of the result space has large connected regions of high values of *MI*, i.e. highly predictable behavior. These connected regions imply that it has little sensitivity to changes in the *execution variables* 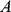 and 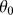. In contrast, in the left chaotic areas, small changes can lead to very different values of *MI*. Hence, a *Sensitivity* measure was defined that quantified the change in *MI*, caused by a unit change in the *execution variables*. This *Sensitivity* was evaluated through changes in the *execution variables* by 10% of their range (due to the different units, normalization was necessary). The ranges of the *execution variables* were: 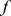: 0.8–1.2 Hz, 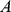: 8–44 cm, 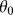: −π/2– +π/2rad, 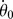: −3– +3rad/s. Accordingly, the 10% change in *execution variables* corresponded to: 0.04 Hz in frequency, 3.6 cm in amplitude, π/10rad in 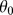, and 0.6rad/s in 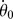. Similarly, the range of *MI* values, 0.006–0.295nat, was used to define the resulting change. *Sensitivity* was defined by the maximum deviation of *MI* for each strategy caused by a 10% change in any of the *execution variables*. Due to the very minor effect of the changes in 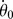 and the high computational costs of these calculations, only the first three *execution variables* were used to compute the *Sensitivity* measure.

#### 2.1.6. Predictability and limit cycle stability: Global Lyapunov Exponent

In one further step of cross-validation of the custom-defined measures, predictability was quantified by the classical measure of the *Global Lyapunov Exponent (GLE)*. The *GLE* could also be considered as a test for the presence of stable limit cycles: positive *GLEs* can rule out the presence of stable behavior [15], [16]. While the previous analysis capitalized on the input-output relation in the system, i.e. on how the desired output correlates with the required input, the definition of *GLE* was based on the kinematic states of the system.

To calculate the *GLE* for each strategy, defined by 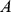, 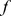, 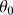 and 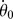, the 4D state-space representation of the system's differential equations was rewritten for ball states in 2D, as the two states of the cup were determined by the desired kinematic and acted as input to the system:
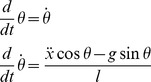
(3)As for the inverse dynamics calculations above, the cup kinematics was assumed to be sinusoidal with known 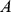 and 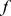. In this formulation, the sinusoidal movement of the cup was represented by 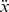. Hence, the resulting ball movement was determined by Equation (3), given the known initial conditions 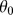 and 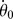. Note that the force applied on the system 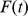 did not directly appear in the equation, and the cup kinematics and the derived ball kinematics sufficed for calculating the *Global Lyapunov Exponent*. As in a state space analysis of the system for trajectory divergence, the Jacobian matrix 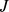 of the state-space representation was used to estimate the *Global (largest) Lyapunov Exponent*:
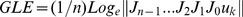
(4)for *n* points along the simulated trajectory, estimated in time-steps of 0.01 s; 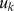 denotes the unit vector (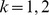), representing the 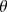 and 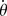 directions [28]. The *GLE* was estimated separately for each of the two unit vectors 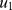 and 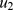. Note that for smooth dynamical systems, the estimation of the *GLE* does not depend on the chosen unit vectors 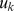 [29]. Nevertheless, we calculated the *GLE* for different choices of 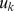. The results did not differ. The numerical integrity of the *GLE* calculations were further confirmed by comparing them against the values of the average *local* Lyapunov exponents determined for the simulated trajectories. Again, the results did not differ. It should be emphasized that this measure of predictability characterizes the object's behavior, not the controller or human's control policy [15]–[17].

**Figure 5:**
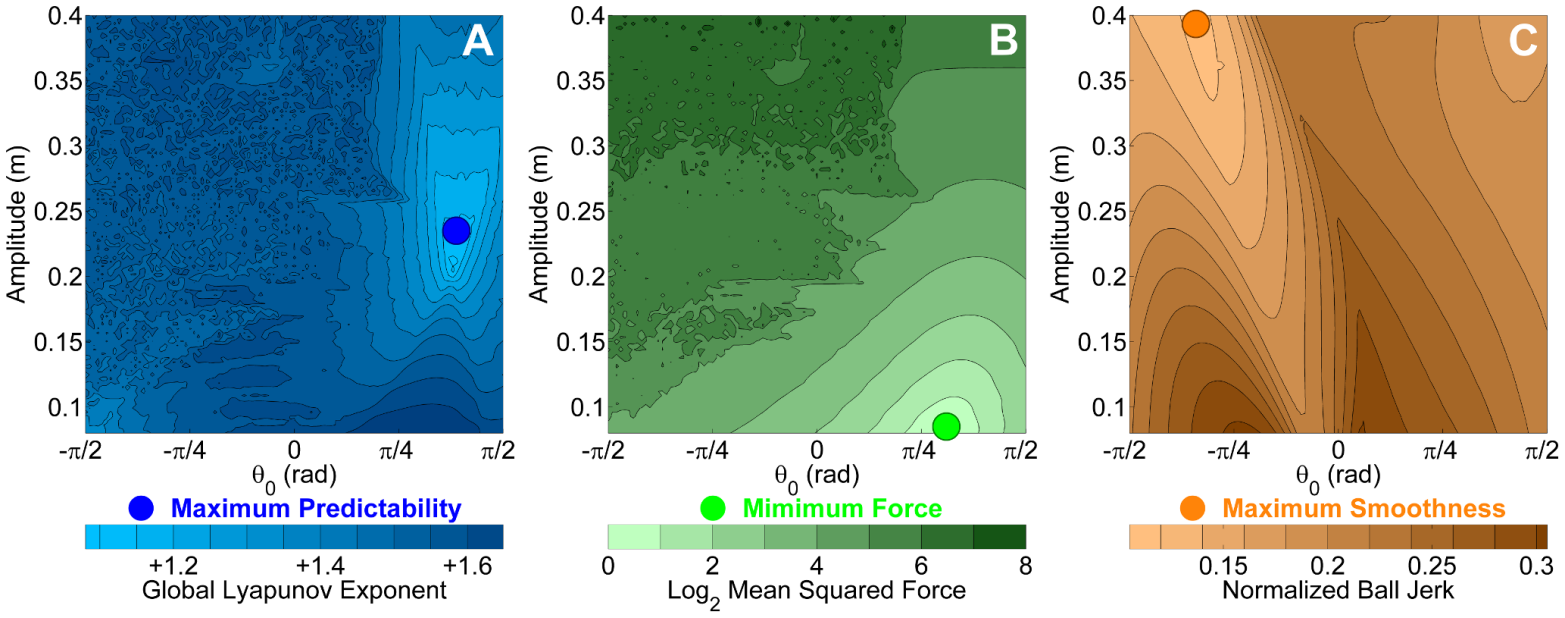
Predicted values in the 2D result space for alternative criteria. **A**: Global Lyapunov Exponent. **B**: Mean Squared Force. **C**: Normalized Ball Jerk. Note that the maps of the Global Lyapunov Exponent and *Mutual Information* are remarkably similar, while the *Mean Squared Force* and the *Ball Jerk* maps predict very different preferred regions in the result space.

The estimates of the *GLE* for each strategy are summarized in the same 2D result space as for *MI*, where two of the four variables were fixed: the frequency was fixed at 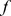 = 1.0 Hz, consistent with the experimental task. Inspection of Figure 5A shows that the smallest *GLE* values were found at 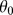 values between π/4 and π/2 rad, i.e. anti-phase behavior. These strategies presented the most predictable behavior corresponding to results in Figure 4. The movement amplitude was very similar to the one for the maximum *MI*. Lastly, the rugged changes of *GLE* in the top left corner, indicative of chaotic behavior, were very similar to the pattern of *MI*. One final important point to note is that all *GLE* values were positive. This implies that all strategies were chaotic, even the one with the smallest *GLE*, hence ruling out the presence of stable limit cycles.

#### 2.1.7. Minimization of effort: Mean Squared Force

Many approaches in motor neuroscience found evidence for minimization of velocity, force, or more generally speaking, effort [18], [19], [30]. To obtain an estimate of the effort exerted for each strategy, the force profile over the duration of one trial run was squared and averaged, to quantify the signal power or *Mean Squared Force (MSF)*:
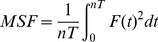
(5)where 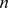 denoted the number of cycles and 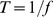 the period of each cycle. Similar to *Mutual Information*, *MSF*-values for all strategies were summarized in the 2D result space. The initial value of the angular velocity 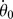 was set to zero for the shown simulation results. However, comparative testing of all initial values of 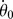 showed that the *MSF* had little sensitivity to different values of 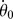.

Figure 5B represents values of the *MSF* (log-transformed for better visualization). The movement strategy with minimum force is found at smaller oscillation amplitudes, where 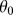 is close to 1 rad (marked by the green circle). This corresponds to anti-phase behavior between the cup displacement 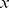 and 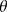. However, unlike *MI*, this strategy would require a very small movement amplitude. The irregular pattern in the top left part of the figure indicates that the boundary from lower to higher values was ragged with islands of smaller force values interspersed. This area again is identified as a highly chaotic parameter region of the system.

To evaluate whether the model system afforded a solution at resonance that required zero force from the subject, linear analysis of the vibration modes was conducted [31]. The goal was to identify the modes of the system and its associated amplitudes. Specifically, the question was whether the system, as parameterized in the experimental task, allowed a resonance mode within the task-specified amplitudes. Linear analysis of the object's free oscillations showed that, besides the free-motion mode, the system's only oscillation mode had anti-phase relation between 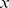 and 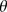 (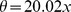); its natural frequency was 1.11 Hz. To find the cup's maximum excursion at the system's free oscillation, we exploited the fact that the displacement of the system's center of mass was always zero in the absence of external force: 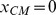. The maximum excursion therefore satisfied: 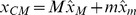. The maximum horizontal distance between the ball and cup was the pendulum's length 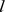, which was the sum of the maximum ball excursion and maximum cup excursion 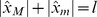. Therefore, the maximum cup excursion was 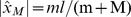 in both positive and negative directions. For the specific parameter values, the maximum theoretically possible cup amplitude in free oscillation was 10 cm.

#### 2.1.8. Maximizing smoothness: jerk

Following the frequently discussed criterion of smoothness in simple reaching movements [21] and the findings by Dingwell and colleagues [10], smoothness or jerk of the trajectories was tested as an alternative criterion that subjects may have optimized. Due to the complexity of the object dynamics, jerk could be minimized in cup, ball, and also force trajectories. In this analysis, only ball and force trajectories were considered, as the cup trajectory was assumed to be sinusoidal and thereby smooth by definition.

To assess the smoothness of ball and force trajectories, the mean absolute jerk was calculated for the simulated profiles of all strategies, defined in the result space by 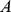, 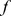, 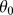 and 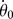. The normalized mean absolute jerk of the ball trajectory was calculated as:
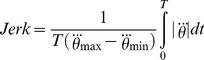
(6)where the ball's jerk was normalized by the ball jerk amplitude to make it dimensionless and, importantly, thereby independent of movement amplitude [32]. Mean jerk was also calculated for the simulated force profiles by replacing 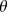 with 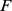 in Equation (6). The measures were calculated with, but also without normalization for verification (see [32] for discussion about normalization and amplitude dependence of measures). As expected, the 2D result space for the non-normalized ball jerk and force jerk predicted the smoothest strategies for the smallest amplitudes. In contrast, when the mean ball jerk was normalized with respect to ball amplitude, the strategies with the lowest jerk values were associated with higher amplitudes. Figure 5C shows the normalized ball jerk in the 2D result space. The orange dot marks the strategy with the highest value for smoothness. The regions with the highest smoothness were associated with in-phase coordination, where 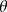 was between –π/4 and –π/2 rad. The mean jerk for the simulated force profiles gave very similar predictions (not shown).

All simulations were performed with the adaptive integration algorithm NDSolve with a precision and accuracy goal of 1e-8 using Mathematica v.8.0.4 (Wolfram Research, Inc., Champaign, IL).

#### 2.1.9. Comparison of predictions and experimental evaluation

Having computed *Mutual Information*, *Global Lyapunov Exponent*, *Mean Squared Force*, and *Jerk* for the strategies in the 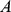 - 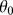 result space, strategies with maximum object predictability, minimum mechanical force, and maximum kinematic smoothness could be identified. Comparison of the maps in Figures 4 and 5 showed different optima and different regions in the 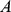 - 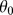 space. The *GLE* and *MI* agreed in predicting highly disjoint, non-smooth, and scattered patterns, signifying chaotic behavior. In the top right region with positive 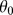 and large 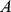 values, the *MI* is high and less sensitive to changes in 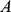. These strategies with high amplitude and anti-phase oscillations are marked with a light blue circle and should be preferred, if predictability of object dynamics is the main criterion for selection. This contrasts with hypotheses derived from *MSF*: in the bottom right region, where 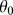 is positive and 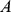 values are small, the *MSF* is at its minimum; predictability of object dynamics is relatively low and *MI* is very sensitive to changes in 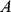 and 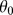. If the mechanical efficiency, e.g. minimization of force, is the main criterion for selection, these strategies with positive 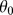 and small 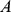 values should be preferred. Finally, the smoothness criterion predicts the negative 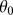 and large 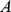 values as optimal, which are associated with chaotic behavior in the predictability measures.

The simulations highlight that movement amplitude was one major distinguishing feature between the hypotheses. Hence, to test what reasons may drive subjects to select certain strategies the experiment did not specify the movement amplitude or ball's motion, but left them free to choose for the subject. Hence, the measured amplitudes will provide a basis to distinguish between criteria. However, the task restricted the allowable movement amplitudes to be between 8 cm and 44 cm to preempt strategies that exploited free oscillations requiring zero force.

### 2.2. Experimental Methods

#### Ethics statement

The experiment was approved by the Northeastern University's Institutional Review Board (IRB#: 10-06-19). Subjects provided written informed consent before participation.

#### 2.2.1. Subjects

Eight healthy right-handed adults (6 female, 2 male, age: 33.8±12) volunteered for the experiment. None of them reported any history of neuromuscular disease.

#### 2.2.2. Experimental setup

**Figure 6:**
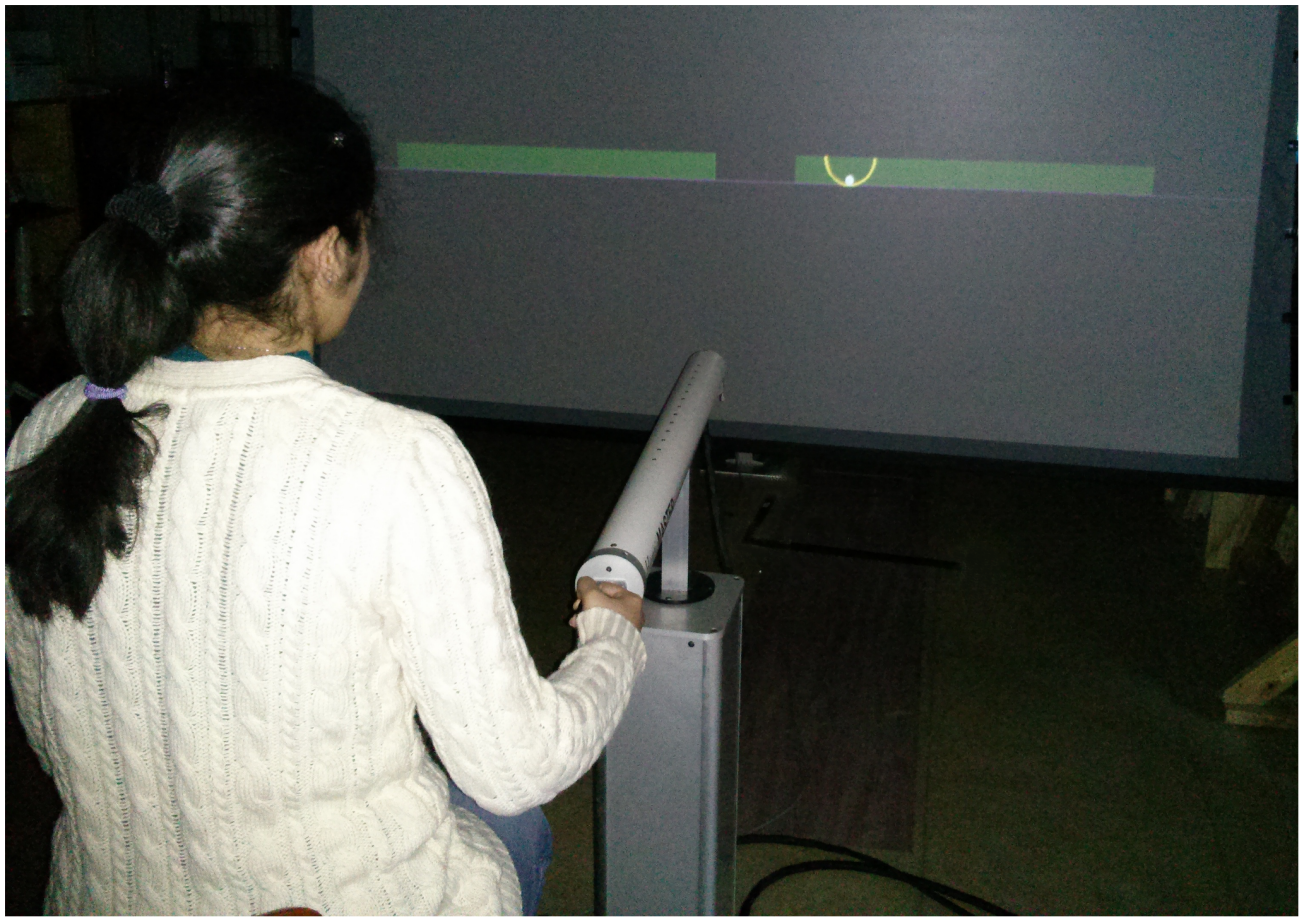
Experimental setup, the robotic arm (HapticMaster), rear-projection screen, and a subject sitting on a chair, while holding the manipulandum of the robotic arm. The task instruction was to move the ball-and-cup object rhythmically between the two green targets at a frequency of 1 Hz. As long as the excursion maxima alternated between the wide target regions, the task was satisfied. Hence, the subjects could choose their movement amplitude.

The dynamics of the two-dimensional ball-and-cup system was simulated in a virtual environment (Figure 6). Subjects manipulated the virtual cup-and-ball system via a robotic arm, which also exerted forces from the virtual object onto the hand (HapticMASTER, Moog FCS Control Systems, Nieuw-Vennep, The Netherlands). Custom-written software in C++ was developed to control the robot and the visual display based on HapticAPI (Moog FCS Control Systems). Subjects sat on a chair and grasped the knob at the end of the robotic arm with their preferred hand to interact with the virtual environment. The sitting position and the distance to the manipulandum were adjusted so that the movements remained within the subject's workspace limits. The virtual task was displayed on a rear-projection screen (2.4 m×2.4 m) that was at a distance of 2.15 m in front of the subject. The visual display consisted of a cup and a ball, shown in white against a black background. The cup was drawn as a semicircle with an arc length of 180 deg, along which the ball moved. The visual display gain between the dimensions in physical displacement of manipulandum and on the visual screen was 4.0. The position, velocity, and acceleration of the cup and ball and the interaction force between hand and manipulandum were recorded at 120 Hz.

#### 2.2.3. Task and protocol

Subjects were asked to move the ball-and-cup system horizontally between two targets in synchrony with a metronome that played auditory stimuli at 2.0 Hz. Subjects were instructed to synchronize the right and left excursions with each metronome sound to achieve an oscillation frequency of 1.0 Hz. If their movements consistently deviated from the metronome pace, subjects were verbally corrected by the experimenter. However, this happened very infrequently after the first few trials. Importantly, the task did not prescribe a specific amplitude, but rather allowed the subject to choose their preferred amplitude in the range between 8 cm and 44 cm. In addition, the instruction explicitly emphasized that respecting the boundaries of targets was not a priority, i.e. it imposed no spatial accuracy demands. Guidance for the range of movement amplitudes was provided by two green target rectangles (Figure 6). As long as the subject reversed the movement within these long rectangles, the task was achieved. The ball could not escape from the cup and the subjects were explicitly informed that they did not have to achieve particular ball motions. Even though the ball could not escape from the cup, the ball motions were shown to provide subjects with visual information about the ball and the forces it exerted onto the hand. We opted to not constrain the ball movements in the instructions (contrary to [1], [10]) because these additional constraints would have interfered and made the task too complex.

Subjects performed 5 blocks of 10 trials each; one trial lasted 45 s. All trials were separated by a 15 s pause and there was a break of several minutes between each block. Subjects could take additional breaks between blocks, if they felt fatigued.

#### 2.2.4. Analysis of experimental data: definition of execution variables

Figure 2 (right) shows examples of the measured kinematic trajectories and forces, including definitions of the experimental variables. As the task instructions elicited trajectories very close to a sinusoid, the cup trajectories were sufficiently described by amplitude 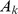 and frequency 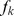, where the subscript denotes the estimate at cycle 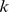. At each peak amplitude of the cup trajectory, the angle and angular velocity of the ball were strobed to obtain 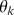 and 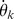. These strobed values at cycles 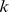 served as estimates of initial states of the ball for each cup cycle. The four variables 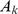, 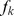, 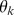, 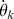 were the experimental analogues to the *execution variables* 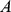, 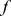, 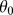, 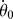 in the model simulations. For each trial, averages of the four *execution variable*s were calculated over 21 cycles in the time window of 25–45 s. The initial 25 s of each trial were excluded, as they frequently contained transient adjustments. Averages were used, because in the experimental trajectories these estimates could take on different values due to changes in the applied force. These average values were viewed to reflect the subject's intended interaction with the object.

Note that the exemplary profiles in Figure 2 are not meant to validate the similarity of simulation and experimental data. While the experimental data show the object's behavior under subject's continuous control, the simulation by definition shows perfect cup sinusoidal trajectory, with no correction. The force profiles differed as the cup profiles were different.

#### 2.2.5. Analysis of experimental data: calculation of strategy measures

To evaluate how the different criteria for object manipulation can account for the results, measures of predictability, force, and smoothness were calculated from the experimental data in two ways. The first method used the average strobed *execution variables* 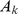, 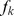, 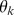, 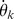 and looked up the corresponding *Mutual Information* and its *Sensitivity*, *Mean Squared Force*, and *Mean Jerk* from the result space calculated by the simulations. Note though that the simulations show the system's behavior in presence of a perfect realization of the sinusoidal cup trajectory, without any intermediate correction for the upcoming cycles. Although this is an idealization, it quantified the predictability of the object, starting from each cycle. Therefore, we also applied a second method that was independent of this assumption and quantified the combined behavior of the object and human's applied control directly from the continuous experimental data.

It needs to be pointed out that in the simulated data, *Mutual Information* reflects predictability based on the chosen initial condition, in perfect execution with no intermediate correction. Evidently, these conditions were not the case in the actual experiment, where subjects may have continuously corrected for deviations. Therefore, the experimental *MI* measure reflects the consistency and repeatability of the subject's performance during interaction with the object. While the simulated measures only represent the object's behavior, the experimental measures characterize the combined effect of the object behavior and the control of the subject during the experiment, i.e. the property of object and the controller together.

To calculate *MI* from experimental data, the continuous force profile and the continuous cup phase 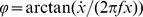 were used in Equation (2), as in the simulations. The calculation of experimental *MI* followed the same procedure as in the simulated *MI*, except that the probability density functions were estimated from continuous experimental data, rather than continuous simulation outputs. To calculate the *Mean Squared Force*, the continuous force profile of each trial was squared and averaged, analogous to the simulated data (Equation 5). To assess the smoothness in the experimental data, both normalized and non-normalized mean absolute jerk were calculated to quantify the smoothness of ball, cup, and force profiles, 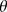, 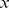, and 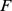. Additionally, the spectral arc-length measure [33], i.e. the arc length of the normalized spectral density function of the signal's time history, was calculated to quantify the smoothness of the experimental data. This measure has been shown to be more consistent in terms of normalization requirements [33]. To characterize the smoothness of the intended/selected strategies rather than the cycle-to-cycle variability, the signal profiles were first averaged across the cycles of each trial and smoothness was estimated on this averaged cycle.

All data analyses were conducted in MATLAB v.2012b (The Mathworks Inc., Natick, MA).

#### 2.2.6. Statistics

To determine the changes with practice in the *execution variables* 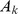, 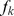,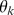, 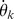 and in the strategy measures, *Mutual Information* and its *Sensitivity*, *Mean Squared Force, and Mean Jerk*, the average values of the first 5 and last 5 trials of each subject were compared by paired *t*-tests as representative of early practice and late practice strategies.

## Results

### 3.1. Execution Variables

**Figure 7:**
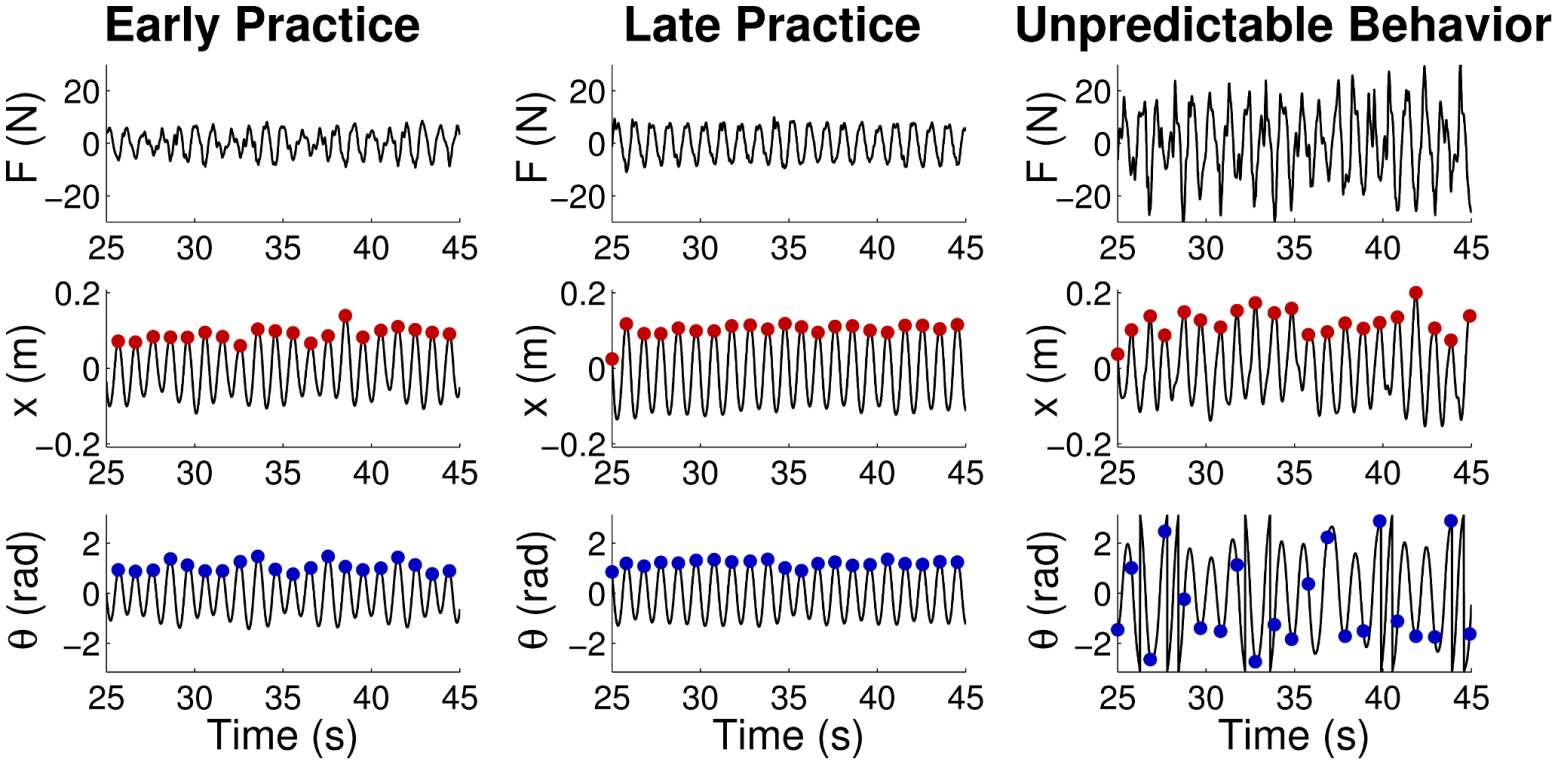
Representative profiles of raw experimental data from early and late practice. 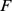
 is the interaction force between the subject and robot, 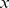 is the displacement of the cup (end-effector), and 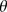 is the angle of the ball. The time of peak cup positions (shown by red dots) served as time reference to strobe the ball angular displacement 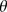 and define 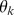. Notice that the relatively irregular patterns of cup displacement and especially force in early practice became more regular and also larger in amplitude later in practice. An example of a highly unpredictable or chaotic behavior is shown on the right. Notice the negative values of 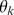, which indicate the chaotic regime, shown in Figure 4. The Mutual Information measure for these continuous experimental data are 0.1256nat (early practice), 0.1693nat (late practice), and 0.1295nat (unpredictable behavior).

Figure 7 shows representative trials of force, cup, and ball displacements, one set from early and one from late in practice. While the cup trajectories were generally rhythmic as required by the task, it is notable that all profiles became more regular with practice. A third set of trajectories shows an example of an unpredictable force profiles and ball movements that were probably highly chaotic. Such chaotic episodes were present in all subjects, but occurred more frequently during early practice. The red dots on the cup displacements at each maximum indicate the strobe points, giving estimates of 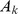 and 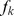, the blue dots on the ball angle indicate the strobed values of 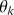 and 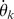 (not shown). These *execution variables* of each trial were averaged to represent the average strategy in each trial (see supporting Figure S2 for an example distribution before averaging for one subject).

**Figure 8:**
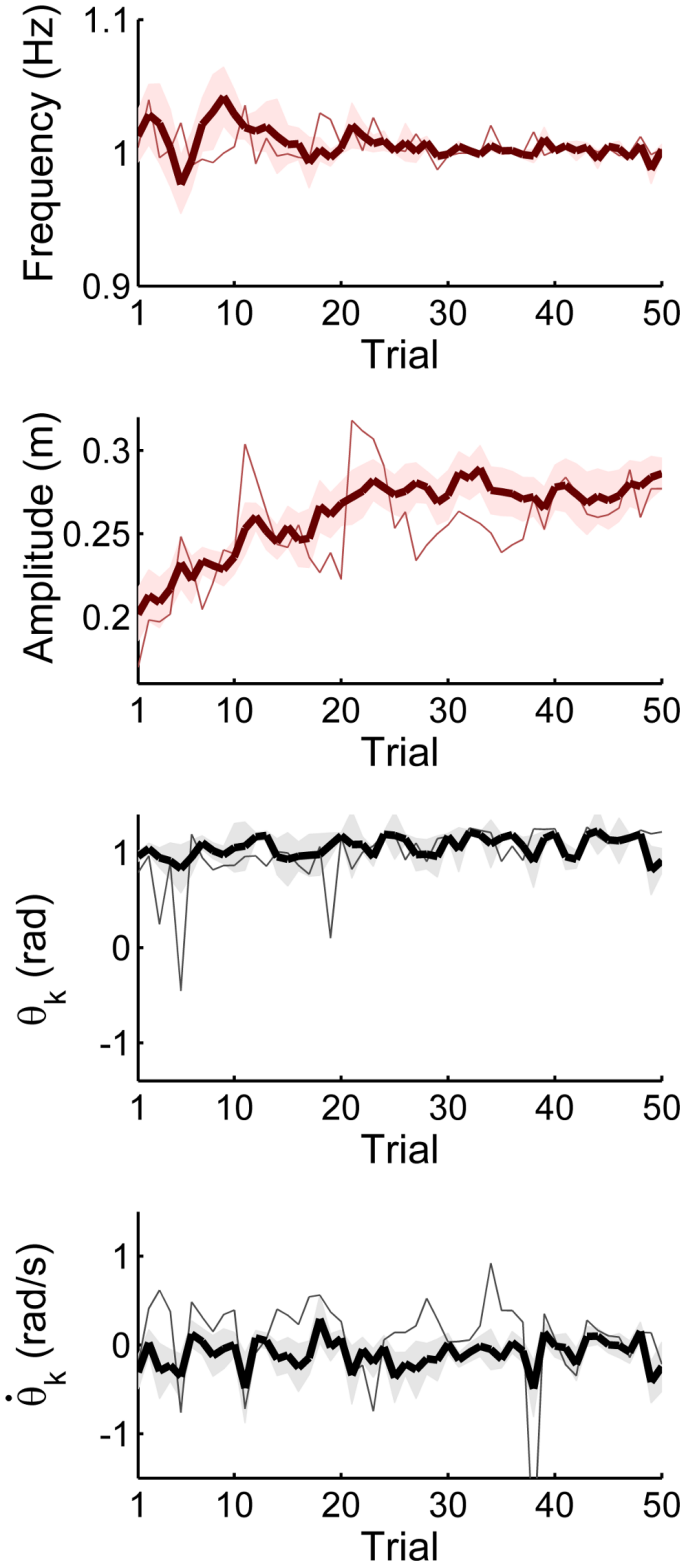
*Execution variables* across 50 practice trials: frequency 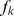 of cup displacement shows that the task was mostly performed at the instructed frequency of 1.0 Hz, amplitude of cup displacement 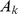 showed a significant increase across practice, ball angle at peak cup position 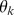 was approximately 1rad, and ball angular velocity 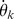 was close to zero throughout practice. The bold lines show the averages across subjects (n = 8); the shaded bands represent one standard error across subject means; the thin lines show one representative subject.

Figure 8 shows how the execution variables develop for all subjects over the 50 practice trials. The four plots show the mean values across subjects with the shaded band representing one standard error around the subject mean. The thin line shows one representative subject. Subjects improved in performing the task at the instructed frequency of 1.0 Hz, as seen by the converging line to 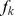 = 1 Hz and the decrease in the standard error, especially after trial 30. The fluctuations across trials also decreased in the single subject, representing the behavior of all other subjects. While the fluctuations within and across trials decreased, the average frequencies in the beginning and at the end of practice were 1.01±0.06 Hz and 1.00±0.01 Hz, respectively, which were not significantly different (p<0.05). In contrast, movement amplitude 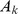 visibly increased with practice, especially until approximately trial 30, from whereon it asymptoted. The average value increased from 21.45±3.86 cm early in practice to 28.03±3.21 cm late in practice, which was significant (p<0.05). The strobed ball angles 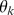 (measured at maximum cup position in each cycle) were positive on average and increased from 0.85±0.33rad in early practice to 0.97±0.24rad in late practice; however, this change was not significant (p>0.05). By coordinate conventions the cup movements to the right and clockwise ball movements (to the left) were defined as positive. Therefore, positive 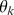 values indicate anti-phase coordination. Trials with negative 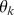, i.e. in-phase coordination, corresponded to strategies in the chaotic regions in Figures 4 and 5. Consequently, performance in these trials was very variable or unpredictable. The strobed angular velocity of the ball 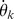 showed a rather inconsistent pattern across subjects, but remained relatively constant across practice. The values at early practice (−0.23±0.48 rad/s) and late practice (−0.11±0.38 rad) were not significantly different (p>0.05). Importantly, the 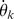 values did not significantly differ from zero, indicating that when the cup was at maximum excursion with zero velocity, the ball was also at maximum excursion with zero velocity. This result confirmed that the assumption 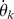 = 0 for plotting the data was acceptable.

### 3.2. Preferred Strategies in Result Space

Although the preferred movement strategies were defined in the 4D result space defined by 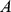, 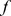, 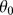 and 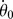, visualization of experimental strategies was possible in the 2D subspace as the frequency was relatively invariant at 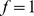
Hz due to the metronome pacing, and the initial cup velocities at maximum excursion were close to zero (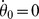). Additionally, 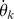 had little effect on the strategy measures *Mutual Information* and its *Sensitivity*, *Global Lyapunov Exponent*, *Mean Squared Force*, and *Mean Jerk*.

**Figure 9:**
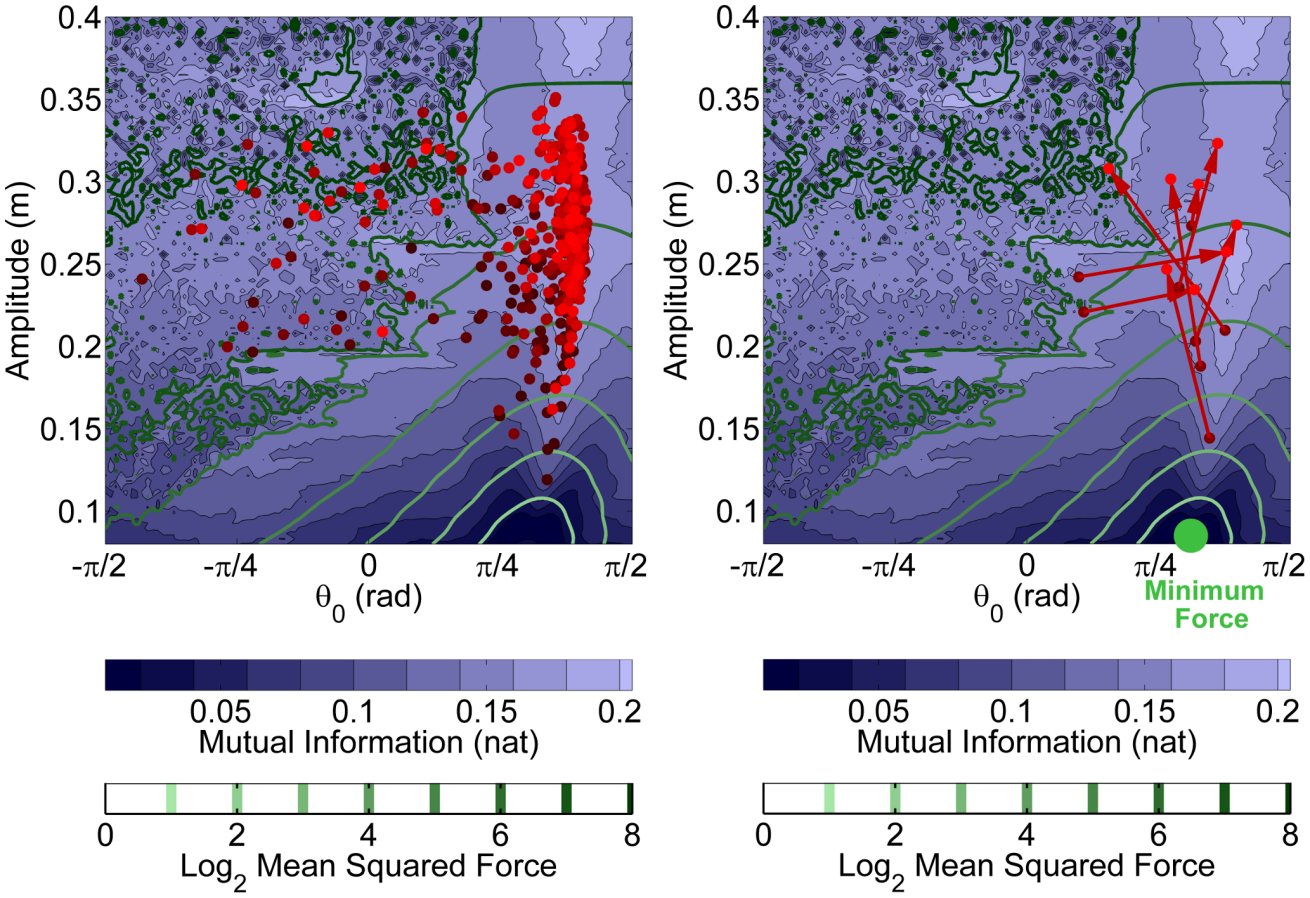
Strategies for all subjects and all trials displayed in the 2D *result space*. The horizontal axis corresponds to the simulation variable 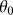 or its average experimental estimates 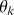 in each trial. The vertical axis corresponds to the simulation variable 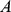 or its average experimental estimates 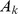. Each point represents the average strategy in one 45 s trial. Darker red indicates early practice and lighter red indicates late practice. The left panel shows that subjects explored regions with lower *Mutual Information* and lower *Mean Squared Force*; however, the majority of trials converged to areas with higher *Mutual Information* and lower *Sensitivity*. The right panel shows the same data separated by subject: the red arrows mark how each subject's average strategy changed from early practice (first 5 trials) to late practice (last 5 trials). All subjects increased their movement amplitude, associated with an increase in overall exerted force. The majority of subjects switched from low- to high-predictability regions in the *result space*. None of the subjects moved toward the minimum force strategy.

Figure 9 shows all trials of all subjects in result space: Each point represents the strategy of one trial. The dark and light color shading corresponds to trials in early and late practice, respectively. The left panels shows that most trials, especially those late in practice, are in the right half of the map, where 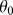 is positive and *MI* shows extended regions of high values, i.e. low *Sensitivity*. The clustering of trials in the regions with higher *MI* is visibly denser. The right panel shows the same data, but now separated by subjects. The arrows connect the average performance in the first and last 5 trials to highlight the change with practice. It can be seen that most subjects shifted to higher movement amplitudes and positive 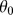 (i.e. anti-phase oscillations), which were associated with higher *MI*. While the individual strategy changes were different, 6 out of 8 subjects increased *MI*, the predictability of interaction; 2 subjects approximately maintained the value, and 7 of 8 subjects decreased the *Sensitivity*. To elaborate, 2 subjects improved the predictability primarily by changes from in-phase towards anti-phase behavior; the remaining 6 subjects, who were at anti-phase behavior from the beginning, increased their amplitude and thereby decreased their sensitivity. This result was consistent with the hypothesis that predictability is a primary criterion for strategy selection. The results simultaneously argue against alternative criteria: None of the subjects moved toward the strategy with maximum smoothness or minimum force (Figure 5).

### 3.3. Mutual Information and Sensitivity, Mean Squared Force, and Smoothness Measures

**Figure 10:**
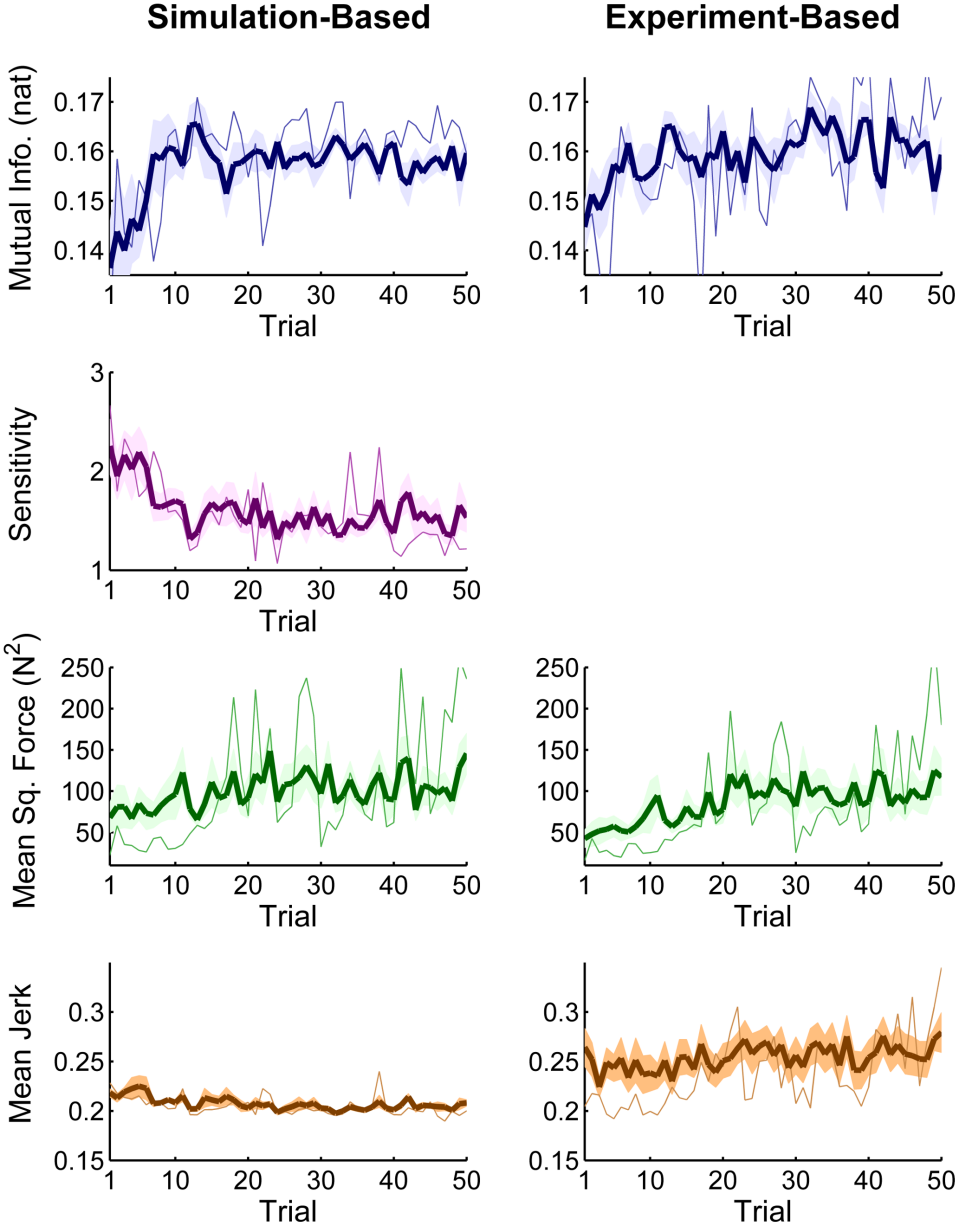
Strategy measures *Mutual information* and its *Sensitivity, Mean Squared Force*, and *Smoothness* plotted across all 50 trials. Thick lines are the across-subject average, the shaded bands show one standard error around the subjects' mean; the thin lines show one representative subject. Left column: model-based strategy measures, simulated at experimental *execution variables* that at each point show the behavior of the object in the upcoming cycle(s) for perfect realization of a sinusoidal cup trajectory. Right column: strategy measures determined from continuous experimental force and kinematics that indicate the combined behavior of the object dynamics and the continuous online control by subjects. Note that *Sensitivity* can only be determined based on the simulated maps.

Figure 10 shows the strategy measures as determined by the two methods: The panels on the left show the measures based on the mean estimated *execution variables* and calculated from the simulated model data; the panels on the right show the strategy measures calculated from the continuous experimental data. The bold lines represent the subject means, the shaded bands indicate one standard error. The thin lines represent a representative subject to show the fluctuations in a single subject.

The top two panels display *MI* between the force profile and cup kinematics (blue). The average values of the simulated *MI* on the left showed a marginally significant increase from 0.142±0.023 nat to 0.158±0.004 nat in late practice (0.10<p<0.05, 6 out of 8 subjects showed an increase). This increase mainly occurred from trial 1 to 13 and then plateaued until the end of practice. The experimental *MI* measure in the right panel showed a similar trend by increasing from 0.1506±0.010 nat to 0.1590±0.007 nat, although this change was not significant (p>0.05). This increase in *MI* shows that object's predictability can explain the changes in strategies.

The panel in the second row shows the *Sensitivity* measure (purple), which could only be derived from the simulated data. The average value decreased significantly from 2.12±0.65 in early practice to 1.5±0.27 in late practice (p<0.05). The change predominantly occurred until about trial 30 and then stayed relatively constant. This decrease in *Sensitivity* was additional support for the hypothesis that object's predictability can account for the change and selection in the manipulation strategies.

For all measures, one individual subject was also plotted to highlight the pattern of variability within a subject. It is noteworthy that in all measures there were relatively large changes across trials, which probably reflects the fact that the subject interacted with a chaotic system. These fluctuations, seen in all subjects, gave rise to the relatively large standard error.

The next two panels (green) display the simulated and experimental average *Mean Squared Force (MSF)* across trials, i.e. the mean squared interaction force within one trial. The simulated *MSF* on the left shows a visible increase from 76.29±48.30 N^2^ in the first 5 trials to 112.71±44.53 N^2^ in the last 5 trials. However, due to the variability, this increase is non-significant (p>0.05). In parallel, *MSF* determined from the continuous recorded interaction force increased from 50.61±31.19 N^2^ in the first 5 trials to 105.88±52.61 N^2^ in the last 5 trials, which was significant (p<0.05). The increase was reached at about trial 30; afterwards, the *MSF* showed little change, similar to the sensitivity measure. Note that the effort-based cost functions predicted that force should be *minimized* with practice, i.e. predicted a *decrease* in exerted force. Hence, both measures of force reject minimum effort strategies.

Figure 10 (bottom row, in brown) shows one of the smoothness measures, *Normalized Ball Jerk*, plotted across the practice trials. Comparison of the first 5 trials vs. late 5 trials across 8 subjects (paired *t*-test, α = 0.10) failed to show a significant decrease. The same comparison was performed for all other *Jerk* measures of the ball trajectory 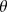, cup trajectory 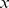, or force profile 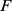, on the non-normalized mean absolute jerk, normalized mean absolute jerk, or spectral arc-length measures. None of the early-late practice comparisons rendered a significant difference.

## Discussion

This study used a virtual task to examine how the complex nonlinear dynamics of an object, specifically the predictability of object dynamics, determines preferred manipulation strategies. Inverse dynamics simulations of the task model rendered insight into what type of forces are required to achieve the task. It is important to emphasize that our approach did not assume any controller. Instead, it calculated the set of all force profiles that generated the task-required cup trajectories for given parameters and initial conditions. This approach is consistent with previous research of our group, where the tasks of throwing and bouncing a ball were analyzed and solution manifolds derived [34], [35]. Determining the manifold of zero-error solutions is comparatively easy for these discrete tasks, where solutions are fully determined by a vector of scalar variables. For example in throwing, the error or task result is a function of position and velocity at release, the *execution variables*. Such a relation becomes considerably more complex, when the task involves continuous interactive control, such as the current cup-of-coffee task. To afford a compact overview over all possible solutions to the task, each continuous strategy was characterized by four scalar *execution variables*: amplitude, frequency of the cup, and the initial or strobed position and velocity of the ball. The resulting continuous trajectories were characterized by different strategy measures or result variables: predictability of the applied force and the kinematic output (*Mutual Information*), and its *Sensitivity* to small changes in the *execution variables*, also expressed in the *Global Lyapunov Exponent*, *Mean Squared Force*, and normalized ball *Mean Jerk*. Model simulations rendered the space of solutions and their result characteristics that served as quantitative criteria to evaluate human manipulation: maximizing predictability of the object dynamics, minimizing the exerted force, and maximizing smoothness.

The experimental task was parameterized to dissociate between the candidate criteria: strategies that maximized predictability were associated with higher forces, while lower forces were coupled with less predictability. Similarly, the strategies that maximized smoothness neither maximized the object predictability, nor did they minimize effort. Thereby, experimental data from humans could dissociate between these criteria.

Results revealed that subjects chose strategies that rendered their interactions with the object dynamics repeatable and predictable, even though these strategies demanded higher force levels and less smooth trajectories. Importantly, these strategies were not stable limit cycles, but positive Lyapunov exponents throughout all strategies indicated chaotic solutions. Over 50 practice trials, subjects' movement amplitudes increased and thereby the measures of predictability increased and also the sensitivity of predictability to *execution variables* decreased. Although the change in *Mutual Information* was only marginally significant, further inspection of the experimental results showed that many subjects already had reasonably high values in early practice and practice only helped them to better maintain it. Additionally, the sensitivity measure showed a significant decrease, lending further support to the hypothesis that predictability played a central role. The measure of exerted force increased significantly across practice. This finding contradicted the dominant role of force minimization in human motor coordination [18], [19], which is frequently invoked as one of the main optimization costs in optimal feedback control [11], [36]. The analyses also highlighted the nonlinearity in this continuous object manipulation task: The result space contained areas of chaotic solutions that were associated with lower predictability, but they were clearly avoided by subjects.

Taken together, these results speak against the possibility to explain the selected strategy based on minimization of force or maximizing smoothness, nor seeking stable limit cycle solutions. Instead, the best account for the observed movement strategies was the notion that humans maximize predictability of object behavior in rhythmic manipulations. Note that in optimal control it is always possible and advantageous to combine cost functions as a weighted sum to arrive at the best possible control solution [37]. While of evident merit in control, this step is scientifically less appealing as this easily becomes unprincipled fitting of data with unlimited options.

### 4.1. Measures of Object Predictability

Evaluation of subjects' movement strategies proceeded along two ways: In a first analysis, the continuous trajectories were strobed to obtain estimates of the four *execution variables* that corresponded to the theoretically derived variables. The average strobed values per trial could be directly mapped into the theoretically derived result space. Then, *Mutual Information* and its *Sensitivity* could be looked up in the theoretical map. Note that these predictions were based on the assumption of perfect realization of sinusoidal cup trajectories, starting from the current states, and excluding any online cycle-by-cycle control of the subject. This simulated condition therefore truly quantified the object's long-term predictability. In real performance, however, subjects constantly applied intermediate online control on the object during the experiment. Therefore, a second analysis was based on the measured continuous trajectories and force and quantified the combined behavior of the object dynamics and the control applied by the subject. The continuous experimental *Mutual Information* showed the same increasing patterns and confirmed the consistency and repeatability of subject's interactive movements.

The map of *Mutual Information* in result space shows connected regions of high predictability that are less chaotic and lead to less unpredictable changes in case of minor changes in amplitude or ball angle. This feature is quantified by the *Sensitivity* measure. Subjects' behavior documents that they not only seek strategies that are predictable, but also those that lower the sensitivity to small deviations in the parameters, corroborating the relevance of predictability. As the analysis of the simulated data rested on the sinusoidal assumption, we also repeated the calculations with sinusoids that had added harmonics of small amplitudes. The results were sufficiently robust with respect to these added perturbations.

Given the centrality of the concept of predictability, the theoretical analysis developed two different measures for its quantification: *Mutual Information* between the kinematic and force profiles quantified to what degree the dynamic behavior differed from simple periodic input-output behavior. A second measure quantified the normalized average variance of the strobed force values as plotted in the bifurcation plot. The map of this *Predictability Index* is shown in supporting Text S1. While not exactly identical, the similarity is striking, given that very different quantifications were used. Hence, their relative congruence can be considered as cross-validation of the *Mutual Information* map. Finally, these input-output measures were verified against a more classic measure based on system's kinematic states, the *Global Lyapunov Exponents*: as expected, the maps of *Mutual Information* and the *Global Lyapunov Exponent* showed very similar patterns. It should be emphasized that the predictability measures do not describe the variability or noise of the kinematics or kinetics at the end-point. A very smooth force and kinematic profiles may lead to an unpredictable relationship; conversely, some complex kinetic and kinematic trajectories with fast fluctuations may form relatively predictable patterns. It is the mutual correspondence of the kinematic and kinetic temporal profiles across consecutive cycles that were captured by the information-theoretic measure, *Mutual Information*.

The pattern of the smoothness measure was visibly different from the predictability and Lyapunov exponent measures. To corroborate these predictions, *Mean Jerk* was calculated for force, ball, and cup profiles and in several different ways: in addition to calculating *Mean Jerk* in the time domain, we also calculated a recently proposed measure for quantifying smoothness: the spectral arc-length in the frequency domain [33]. Ball trajectory and force profile variants of smoothness measures (regardless of normalization) yielded very similar surfaces in the 2D result space. Comparison of the average experimental measures in early and late practice showed no significant decrease for any of the 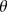, 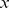, and 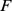 by either of the smoothness measures. This further supported that subjects did not seek to maximize smoothness in their preferred movements.

### 4.2. Alternative Explanations and Criteria

In the literature on unconstrained movements numerous other optimization criteria have been proffered as viable candidates for motor control. However, as this study shows, physical interaction introduces very specific challenges that potentially override other criteria that may be relevant for unconstrained motion. We will discuss potential candidates in turn.

#### 4.2.1. Temporal accuracy

As the task involved synchronization with the metronome, one potential alternative aspect that may have affected the outcome is the subjects' goal to achieve temporal accuracy and synchrony with the metronome. Note though that this criterion was not emphasized and there was no quantitative feedback on timing accuracy. Subjects were only reminded of this criterion, if they consistently deviated, which only happened once or twice for each subject. Also, different to typical tapping synchronization tasks, the haptic feedback was not limited to the maximum excursion to serve as a salient landmark for synchronization, such as in tapping movements. Instead, the interaction forces were continuous and presented complex fluctuating patterns. Yet, it may be conjectured that the increase in amplitude was in service of optimizing temporal accuracy. There is some evidence for this correlation in studies on point-to-point movements, where small movement amplitudes could negatively affect temporal accuracy [38]. Further, timing variability was shown to decrease with movement velocities and increase with movement distance [39]. However, these results were reported for discrete pointing or tapping tasks. The present task was continuously rhythmic, without a salient touch or contact event to be synchronized. Discrete and rhythmic movements have shown to lead to very different effects [40]–[43], probably due to different neurophysiological control mechanisms [44]. Hence, the extrapolations of these results are potentially problematic.

In a study on free rhythmic finger movements, no difference in the preferred amplitude was found across various un-paced and paced conditions [45]. This was further supported by a more recent study, where the arm movement amplitude had no effect on the timing error variance [46]. Nevertheless, we analyzed our data to test the hypothesis that temporal accuracy and movement amplitude were positively related. To this end, the temporal difference between each metronome beep and the maximum and minimum excursion of the cup trajectory was determined. To eliminate transients in each trial, only the cycles in the window between 20 to 45 s were used. Correlating the average temporal error with the mean movement amplitude of each trial did not show any significant relation when all subjects' data were pooled, *r* = −0.064, *p* = 0.20. Similarly, correlating the standard deviations of the asynchrony error in each trial with the average amplitude did not yield any significant correlations, *r* = −0.019, *p* = 0.70. Repeating the correlations for the same two asynchrony measures for each individual only showed significance for one subject for the mean asynchrony error. The same results were obtained, even when the analyses was limited to the final 20 trials, after practice effects had subsided.

Nevertheless, two additional small experiments were conducted that probed whether synchronization may have been facilitated at higher amplitudes. One test presented a small and a large amplitude target to subjects, while again paced at 1 Hz frequency. Comparing the temporal error in small and large amplitude conditions showed no difference, i.e. no advantage for synchronization at the larger amplitudes. A second experiment examined subjects' amplitude preference when manipulating a solid object in synchrony with the metronome, i.e. when the complex dynamics were removed. Subjects showed a spectrum of amplitudes between 13 and 32 cm, with the mean at 22.4±6.1 cm. This amplitude was significantly lower than the preferred movement amplitude during late practice of the experiment proper (p<0.05, two-sample *t*-test). Further, the temporal error was insensitive to the movement amplitude. For more details on the experiments see supporting Text S2. Taken together, these findings did not support the hypothesis that temporal accuracy or precision were responsible for the observed amplitude increase. Rather, they confirmed that the object dynamics was the reason for the observed amplitude increase.

#### 4.2.2. Free oscillation modes and stable limit cycles

A complex dynamic system has free oscillation modes that may afford an efficient solution to the task: the free oscillation mode requires zero applied force. It may be conjectured that subjects adjusted their amplitudes to seek this efficient mode to satisfy the task. Indeed, linear analysis of the object's free oscillations [31] showed that the system has a free-motion mode (natural frequency is zero) and an anti-phase oscillation mode with a natural frequency of 1.11 Hz (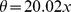). However, given that the center of mass of the system does not move in free oscillation, the maximum theoretically possible amplitude of the cup was 10 cm (assuming the nonlinear characteristics of the system allowed stable rhythmic movements). This was considerably different from the experimentally observed amplitudes at any stage of the experiment and rejected the possibility that the selected amplitudes were dictated by the free oscillation modes. This result is underscored by the fact that according to the *Global Lyapunov Exponent* map, the positive values in the entire 2D result subspace show that none of the strategies generated stable limit cycles. This precludes explanations of the results in terms of stable modes of coupled oscillators [47]–[51].

#### 4.2.3. Preference for frequency-amplitude combinations dictated by stable dynamics

It may be questioned if selecting a higher amplitude was dictated by “comfortable” frequency-amplitude combinations due to mechanical properties or neural control preference [47], [52]. The literature on rhythmic movements has examined the frequency-amplitude relationship in stable paced and un-paced movements. However, the range of preferred amplitudes for a given frequency is typically quite high (e.g. [47], [48], [52]). In addition, observed increases or decreases in mean amplitudes were associated with slower or higher frequencies, respectively. This is congruent with keeping peak velocity at reasonable values and to keep “effort” low. Our data show an amplitude change in the opposite direction: for an invariant frequency, amplitude is increased, which increases peak velocity and effort.

#### 4.2.4. Kinetic and energetic criteria

Choosing larger amplitudes and forces implicitly ruled out that subjects minimized torque change and energy, as both criteria increased simultaneously with movement amplitude and force. The force iso-contours in the 2D result space in Figure 9 also showed that the regions with larger amplitudes required higher forces. Interestingly, however, subjects reported that their acquired strategies felt “easier” or less forceful than their initial strategies. Unexpected interaction forces required corrections based on feedback. Apparently, this additional processing was perceived as more demanding. This observation underscored that the *perceived* effort was smaller in the predictable interactions.

#### 4.2.5. Exploration during practice

It may be speculated that subjects chose the observed strategies, because they were the most trivial or available ones and not because of preference over the other alternatives. It is important to highlight that subjects did explore different parts of the result space. This can be seen in Figure 9, where individual trials were scattered across the result space, especially in the early stage of the experiment. This indicates that the strategies were not chosen due to limited chance to discover them. Also, the relatively negligible change in average strategies from trial 35 to 50 shows that the selected strategies were relatively consistent and preferred, as opposed to a randomly visited strategy.

### 4.3. Information Processing in the Sensorimotor Control System

Rhythmic coordination has been readily associated with stable limit cycle behavior and a lot of support has been accumulated in bimanual coordination. For example, two-finger coordination has been extensively examined and parametric variations in the experiment, such as frequency scaling, have supported predictions from coupled oscillator models [51], [53], [54]. A preference for stable perception-action dynamics has also been shown in a rhythmic ball bouncing task, where subjects preferred movement solutions with dynamic stability [35], [55], [56]. In this task, the dynamically stable solution is attained when the racket impacts the ball at the decelerating segment of the racket trajectory, assuming sinusoidal racket movements. Importantly, this solution was chosen over the mechanically more efficient solution of hitting the ball at maximum velocity of the racket trajectory. Such dynamically stable solutions can be interpreted as computationally less demanding, as small perturbations do not require continuous corrections, but die out by themselves. The same argument can be made for the present task: the strategies with predictable object behavior can be considered as the ones that avoid uncertainty [57] and therefore require little or no error corrections.

The surprising observation in the present task was that rhythmic performance did not appear to be stable, as inferred from positive Lyapunov exponents. These results on stability were corroborated by their match with the predictability estimates. Apparently, the added complexity from the object dynamics introduced chaos, at least in the mathematical model. Hence, subjects chose solutions to the task that offered a surrogate to stability – predictability. Similar to the argument in the ball bouncing task, it can be argued that this eases the demands on information processing, while at the same time increasing the cost of energy processes.

**Figure 11:**
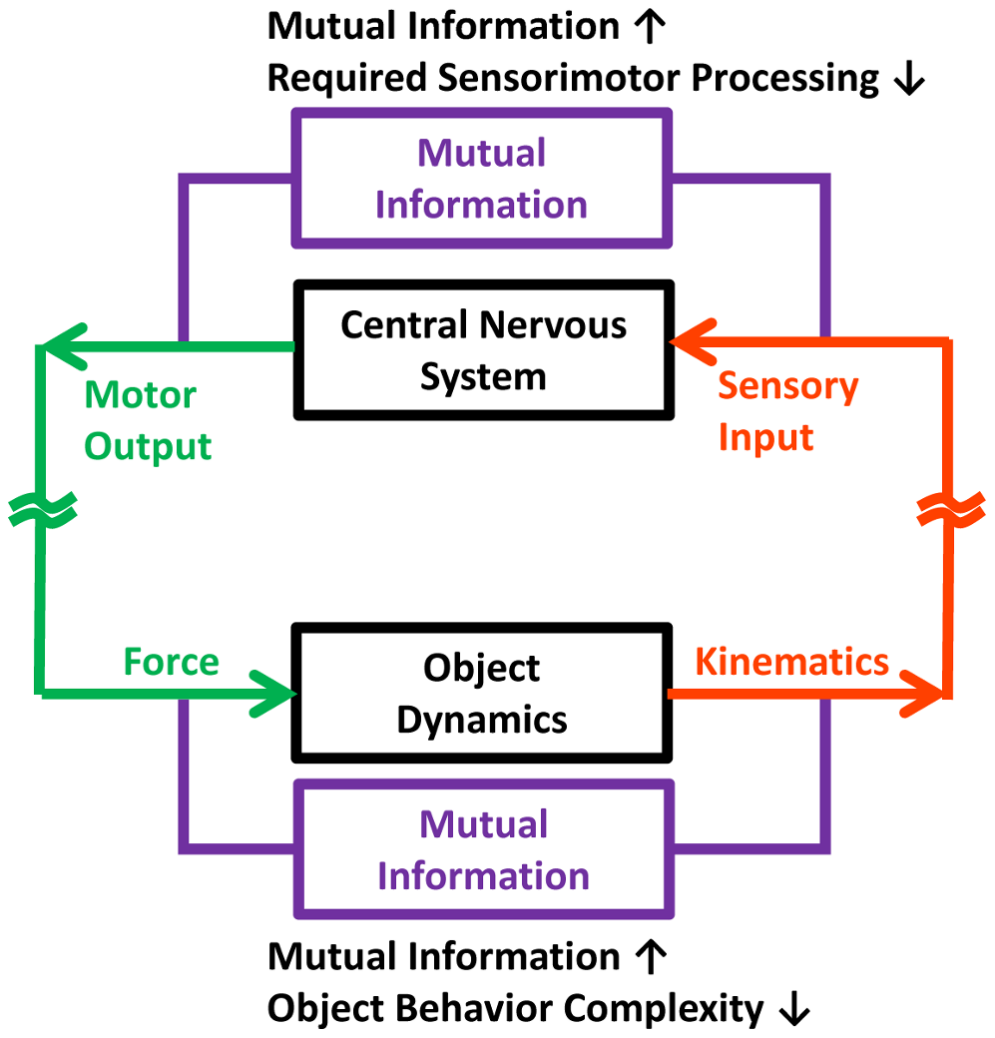
Schematic of the control loop between the central nervous system and the object dynamics. *Mutual Information* between the force and kinematics quantifies the complexity of the object dynamics. At the same time, *Mutual Information* between the sensory information and motor commands quantifies the level of the required sensorimotor information processing. This can be interpreted as a reflection of the complexity of the sensorimotor information processing required by central nervous system for performing the movement task.

Figure 11 schematizes this information-theoretic interpretation and its relation to the processing demands of a sensorimotor control system. If the forces result from motor commands and the object kinematics and dynamics are translated into sensory information, the complexity of the object dynamics can be interpreted as the degree of required sensorimotor processing. *Mutual Information* between the force and the object kinematics quantifies the input-output relation of the system. The less predictable this sensorimotor information mapping, the more demands arise for the controller. In contrast, predictable interactions require less active information processing. This interpretation is consistent with the finding that during rhythmic movements fewer cortical and subcortical areas are activated compared to discrete movements, as revealed by fMRI [44]. It is interesting to contrast this information-processing efficiency with mechanical efficiency, as an extension of one-way channel capacity [58], [59] to a closed-loop information-theoretic measure of sensorimotor control (see also [60], [61]).

#### 4.3.1. Predictability as a control policy

Having demonstrated that predictability is a priority for human control, the question arises how to translate this choice into a control policy. At this point, only speculations are possible. To begin, continuous “surprises” from the interaction are perceived by subjects as cumbersome and hard to counteract. Therefore, the computational cost increases. One possibility is to include Local Lyapunov Exponents or similar measures in the controller's cost function. While this is intuitive, a mathematical formulation can be more challenging, given the potential complexity of the nonlinear dynamical system.

#### 4.3.2. Object characteristics vs. controller characteristics

It needs to be underscored again that in contrast to many other computational studies, the purpose of the simulations was not to model the neural control system at every time-step. Many previous studies investigated models of potential controllers [5], for example to study the role of delays and noise, or the continuous vs. intermittent nature of control [7]–[9]. These studies sought to identify the details of the human control mechanism; hence, they have often simplified the object dynamics to facilitate focus on the complexities of the controller. Our study shifted this emphasis: maintain the nonlinearity and complexity of the object dynamics and identify the strategies that are preferred for control, before modeling the controller and the mechanisms of its neuromechanical implementation [8].

### 4.4. Conclusion

This study highlighted that interactive tasks with complex object dynamics may have solutions with different degrees of predictable or chaotic object behavior. Experimental results supported the hypothesis that predictability of object's dynamics and, hence, interaction between the human and object, is the primary criterion for strategy selection. Quantification of the predictability of the human-object interaction expresses that humans seek solutions where force and kinematics are synchronized into a repeatable pattern, which most likely has lower demands on sensorimotor information processing.

## Supporting Information

Figure S1 Maps of the *Predictability Index* (left) and *Mutual Information* (right). The color maps show that realization of a 1.0 Hz sinusoidal cup trajectory with different amplitudes and different initial ball angles yielded different predictability of the object dynamics. Note that the maps of the *Predictability Index* and *Mutual Information* are remarkably similar.
(TIF)

Figure S2Experimental *execution variables* 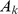, 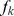, 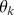, 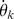 and strategy measures (*Mutual Information* and *Sensitivity, Mean Squared Force*, and *Mean Jerk*), plotted for all cycles of all trials for a representative subject 1.(EPS)

Text S1 Calculation of the force values in the bifurcation diagram and *Predictability Index* of object behavior.
(PDF)

Text S2 Control experiments to assess the role of timing accuracy or precision demand in selected strategies.
(PDF)
