# Supplementary figures and images for: Rhythmic Manipulation of Objects with Complex Dynamics: Predictability over Chaos
Source: PLoS Comput Biol. 2014 Oct 23;10(10):e1003900. doi: 10.1371/journal.pcbi.1003900 (PMC4207605; doi:10.1371/journal.pcbi.1003900)

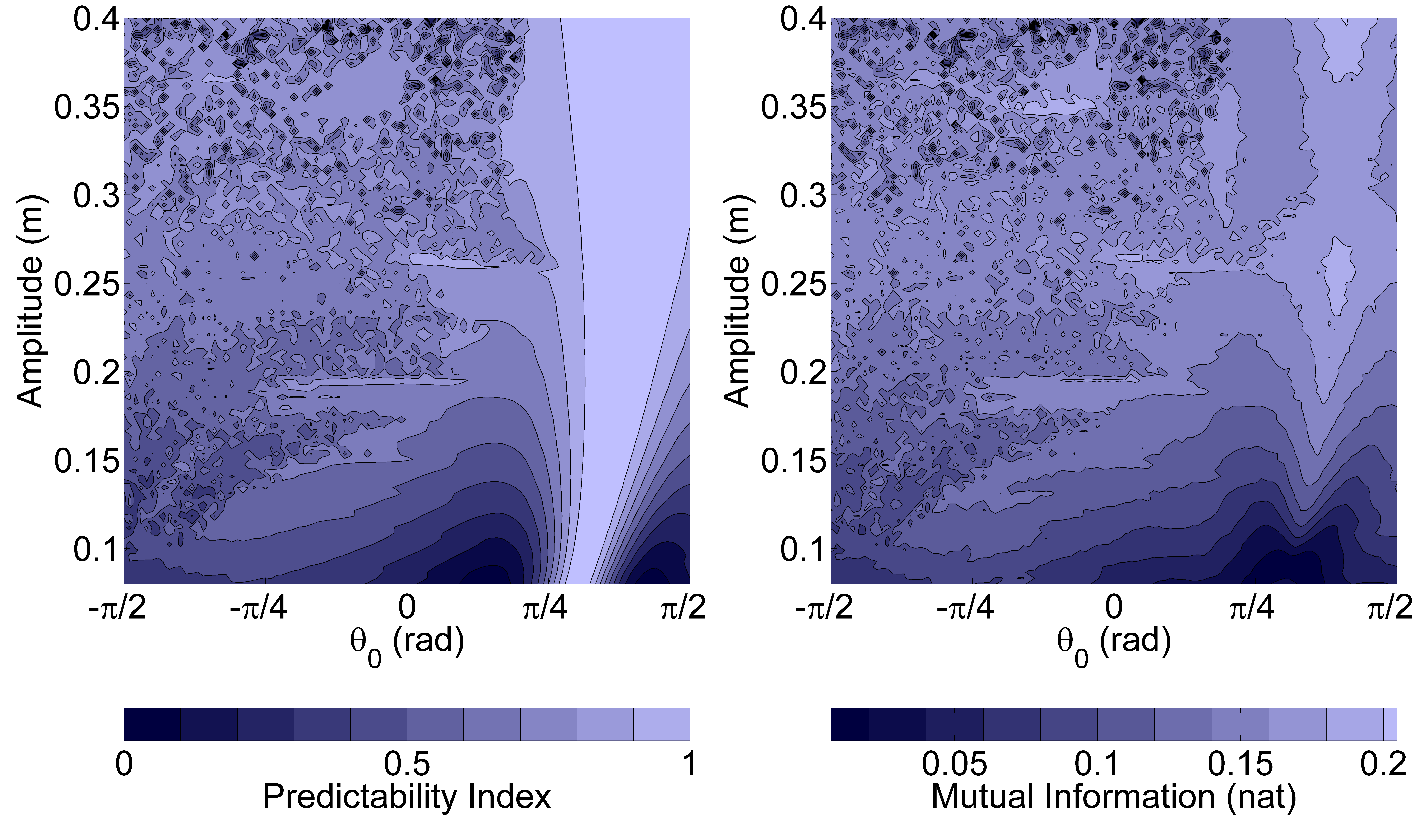

Supplement: Figure S1 — Maps of the Predictability Index (left) and Mutual Information (right). The color maps show that realization of a 1.0 Hz sinusoidal cup trajectory with different amplitudes and different initial ball angles yielded different predictability of the object dynamics. Note that the maps of the Predictability Index and Mutual Information are remarkably similar. (TIF) [file pcbi.1003900.s001.tif]
