# Supplementary material for: Rhythmic Manipulation of Objects with Complex Dynamics: Predictability over Chaos
Source: PLoS Comput Biol. 2014 Oct 23;10(10):e1003900. doi: 10.1371/journal.pcbi.1003900 (PMC4207605; doi:10.1371/journal.pcbi.1003900)
Supplement: Text S1 — Calculation of the force values in the bifurcation diagram and Predictability Index of object behavior. (PDF) [file pcbi.1003900.s003.pdf]

# Supporting Text S1

## 1. Calculation of the Force Values in the Bifurcation Diagram

In mathematical notation, the strobed force distributions were obtained as follows: The cup trajectory $x(t)$ approximated by a sine wave with period $T = 1/f$ and amplitude values between $\pm A/2$ running for $n$ cycles is defined as:

$$x(t), \quad -A/2 < x(t) < A/2, \quad x(t) = x(t+T), \quad 0 < t < nT \tag{A1}$$

The cup displacement value at cycle $k$ and time $\tau$ (which is the cycle time starting at 0 and ending at $T$ ) can be written as:

$$x_k(\tau) = x(\tau + (k-1)T), \ \ 1 \le k \le n, \ \ 0 \le \tau < T \tag{A2}$$

Due to the sinusoidal assumption for $x(t)$, the value of $x_k(\tau)$ only depends on $\tau$ and not on $k$. The force value corresponding to the $k^{th}$ cycle at time $\tau$ can be written as:

$$F_k(\tau) = F(\tau + (k-1)T), \ \ 1 \le k \le n, \ \ 0 \le \tau < T \tag{A3}$$

In contrast to $x_k(\tau)$, the force $F_k(\tau)$ depends both on $\tau$ and $k$ due to the nonlinear dynamics of the system. With this mathematical notation, the strobed force values were defined and displayed in Figure 3. Strobing the force profiles at maximum cup position, $x_k(0) = +A/2$, for consecutive simulation cycles (n = 50) results in the scattered patterns of $F_k(\tau)$ at $\tau = 0$.

## 2. Predictability Index of Object Behavior

To test the reliability of *Mutual Information* as an index for predictability, a second measure was defined on the basis of the strobed force values. This *Predictability Index* was based on the normalized average variance of visited output values at each instance of the cycle. Zero variance denotes perfect predictability and larger values mean less predictability. To estimate the predictability based on one complete cycle, the variance at each point of the cycle was averaged for all the points in the cycle, i.e. for all $\tau$ ‘s. Normalization by the mean squared force values makes this measure independent from the

magnitude, scale, or unit of force and makes comparisons between different tasks and conditions viable. It is expressed as:

$$Predictability\ Index = 1 - \frac{\int_0^T \sum_{k=1}^{n} (F_k(\tau) - \frac{1}{n}\sum_{k=1}^{n} F_k(\tau))^2 d\tau}{\int_0^T \sum_{k=1}^{n} F_k^2(\tau) d\tau} \quad \text{(A4)}$$

The *Predictability Index* has a minimum value 0 as the least predictable condition and a maximum value of 1 as the most predictable behavior. It should be noted that this variance is a cycle-by-cycle variability and differs from the high frequency noise component, usually referred to as variability of force or kinematics. Figure S1 shows the map of *Predictability Index* in the 2-dimensional subspace of the *result space*. Comparison to the map for *Mutual Information* shows that the predictions are remarkably similar.
