# Supplementary material for: Rhythmic Manipulation of Objects with Complex Dynamics: Predictability over Chaos
Source: PLoS Comput Biol. 2014 Oct 23;10(10):e1003900. doi: 10.1371/journal.pcbi.1003900 (PMC4207605; doi:10.1371/journal.pcbi.1003900)
Supplement: Text S2 — Control experiments to assess the role of timing accuracy or precision demand in selected strategies. (PDF) [file pcbi.1003900.s004.pdf]

Supporting Text S2

To further test the hypothesis that synchronization was not an alternative explanation of our results, we conducted two additional experiments. In the first experiment, we tested rhythmic movements with our experimental set-up, where the ball was fixed, i.e. the complex dynamics was eliminated. All other task parameters were kept the same. As in the experiment proper, the movement amplitude was free to choose, and the same rectangular targets were presented; subjects were paced by a metronome to the same 1Hz frequency. Six subjects performed 10 trials (45 sec) each (note this task is very easy and does not require any practice). The question was what amplitude subject would choose and whether the timing accuracy and precision correlated with the amplitude. Determining the temporal difference between the metronome onset and the maximal excursion yielded the timing error, both means and standard deviations.

As the Figure A1 shows, subjects moved at 1 Hz, in synchrony with the metronome. Further, different subjects chose different amplitudes. The average movement amplitude in trials 6 to 10, i.e. the last 5 trials, was 22.4 ± 6.1cm. This was significantly lower than the selected movement amplitude in the ball-and-cup experiment (2-sample t-test, p = 0.04). One out of 6 subjects reduced their amplitude across trials, potentially to reduce effort. None of the subject increased their amplitude.

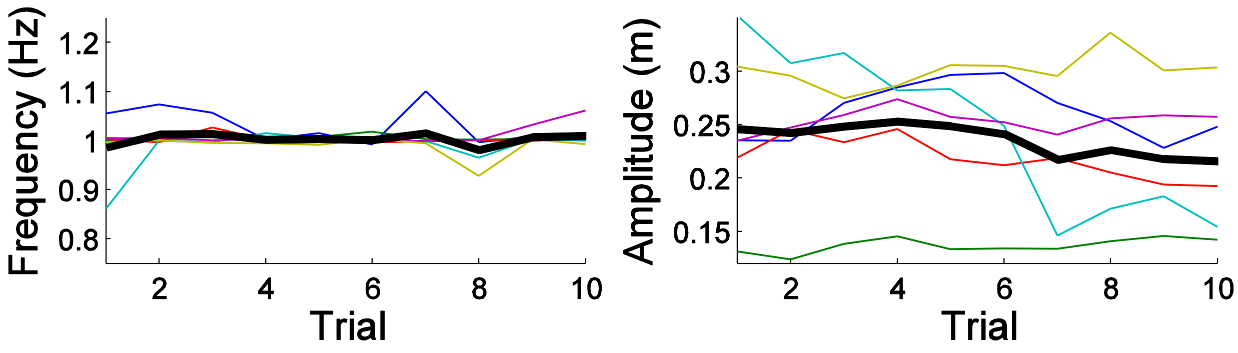

Figure A1. Movement frequency and amplitude in 1Hz rhythmic movement of a solid mass.

Second, correlating amplitude with temporal error and its standard deviations did not render any significant correlation: constant error (accuracy) vs. amplitude: r = -0.05, p = 0.71; variable error (precision) vs. Amplitude: r = -0.04, p = 0.75.

A second experiment was conducted that kept the complex dynamics the same as in the experiment proper. To test whether larger amplitudes afforded better synchrony with the metronome, two target amplitudes were specified: one target at 25cm and a second target at 40cm. These two amplitudes are approximately the range limits seen in the experiment proper. 4 subjects performed a 4 blocks of 5 trials each, alternating between small and large amplitudes, in counterbalanced order. For the last two blocks absolute timing error was determined (mean and standard deviations per trial) and correlated with the mean amplitude in the same trial. As Figure A2 summarizes, the correlations, pooled over all subjects, were not significant: constant error (accuracy): $r = -0.20$, $p = 0.21$; variable error (precision): $r = 0.16$, $p = 0.31$.

When the same correlation was conducted within each individual, only 1 out of 4 subjects showed negative correlation between amplitude and accuracy, while 3 other subjects showed no significant p-values. For precision none of the 4 subjects showed significant correlations between amplitude and precision. The figure identifies each subject by one color.

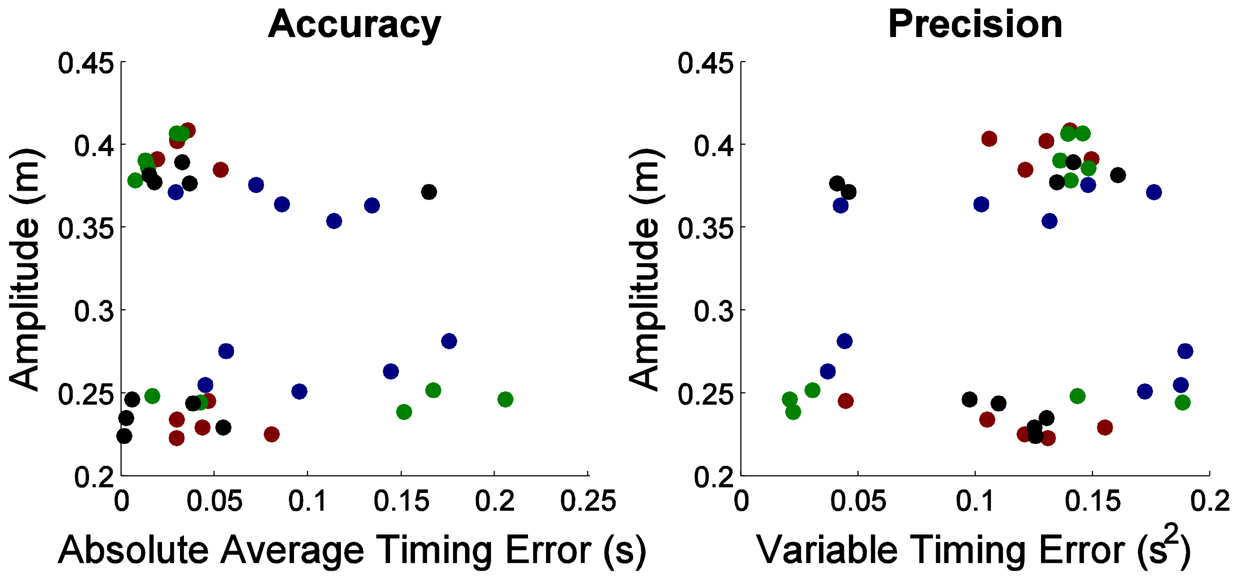

Figure A2. Accuracy and precision of 1Hz rhythmic movement compared at two different amplitudes
